# Effects of the Sex Steroid Hormone Estradiol on Biofilm Growth of Cystic Fibrosis *Pseudomonas aeruginosa* Isolates

**DOI:** 10.3389/fcimb.2022.941014

**Published:** 2022-07-13

**Authors:** Jiwar Al-Zawity, Faria Afzal, Aysha Awan, Daniela Nordhoff, Alexander Kleimann, Daniel Wesner, Tristan Montier, Tony Le Gall, Mareike Müller

**Affiliations:** ^1^ Physical Chemistry I and Research Center of Micro- and Nanochemistry and (Bio)Technology (Cμ), Department of Chemistry and Biology, University of Siegen, Siegen, Germany; ^2^ INSERM, Univ Brest, EFS, UMR 1078, GGB-GTCA, Brest, France; ^3^ CHRU de Brest, Service de Génétique Médicale et de Biologie de la Reproduction, Centre de Référence des Maladies Rares “Maladies Neuromusculaires”, Brest, France

**Keywords:** estradiol, *Pseudomonas aeruginosa*, biofilm growth, cystic fibrosis, clinical isolates, quorum sensing

## Abstract

Women with cystic fibrosis (CF) have a significantly lower life expectancy compared to men, which is indicated by an earlier impairment of lung function due to chronic colonization with biofilm formed by *Pseudomonas aeruginosa*. There is growing evidence that blood serum concentrations of the steroid sex hormone estradiol (E_2_) correlate with the occurrence of pulmonary exacerbations in CF but also play a role in the mucoid switch of *P. aeruginosa*. This study aims to shed light on possible microbiological reasons for sexual dimorphism in CF by investigating the influence of E_2_ on biofilm formation of *P*. *aeruginosa* CF isolates. For this purpose, 10 CF isolates of the respiratory tract derived from different CF patients have been treated with E_2_ in a microtiter plate biofilm model. Biofilms have been examined by crystal violet assays, field emission scanning electron microscopy (FE-SEM), 3D laser scanning microscopy (LSM), and quorum sensing (QS) reporter assays of the supernatants taken from biofilms. This allowed us to simultaneously investigate the effects of E_2_ on attached biofilm mass, biofilm ultrastructure, and QS activity. Upon E_2_ treatment, six out of 10 investigated CF isolates showed an increase of attached biofilm mass, whereas biofilms from two tested non-CF laboratory strains (PAO1 and ATCC19660) did not. Moreover, FE-SEM and 3D LSM analyses of the E_2_ responsive CF biofilms revealed ultrastructural remodeling of biofilm structure at different scales with increased formation of prominent biofilm spots, enhanced coverage with extracellular polymeric substance (EPS), and extended average surface roughness. QS activity measurements performed in biofilm supernatants *via* luminescence acyl homoserine lactone (AHL) reporter assays further showed that E_2_ treatment may also modulate QS signaling, as shown in an E_2_ sensitive CF isolate. Together, our results suggest the biofilm modulating effects of E_2_ on various clinical CF isolates that are documented by both biomass and ultrastructural changes of biofilms. The gained new insight into the influence of steroid hormones on *P. aeruginosa* biofilm phenotypes might pave the way for novel future approaches in personalized medicine based on the patients’ sex and hormonal status.

## Introduction

CF is a hereditary orphan disease that affects 70,000 people worldwide. It is characterized by the production of a viscous mucus, especially in the lung, and caused by a mutation in the Cystic Fibrosis Transmembrane Conductance Regulator (CFTR) gene resulting in malfunction of the chloride channel encoded by this gene. The CF mucus offers perfect conditions for the colonization of biofilm-forming lung pathogens such as *P. aeruginosa*, which can lead to life-threatening lung infections and destruction of respiratory airways (Bhagirath et al., 2016; Bell et al., 2020). Although *Pseudomonas* lung infections have been gradually reduced over the past 10 years, still more than 50% of adult CF patients at 30 years of age exhibit a chronic colonization with *P. aeruginosa* according to recent CF registry reports from UK, Germany, and US (Cystic Fibrosis Trust, 2020; Nährlich et al., 2020; Cystic Fibrosis Foundation, 2021).

Several studies report on a prominent sexual dimorphism in chronical respiratory diseases associated with colonization and persistence of bacterial pathogens. These differences between women and men in CF patients and patients with non-CF-bronchiectasis apply to disease prevalence, severity, and outcome that means an earlier impairment of lung function in women (Vidaillac et al., 2018). Moreover, women have a significantly lower life expectancy than men, a phenomenon that has been defined as the ‘CF-Gender Gap’ (Harness-Brumley et al., 2014; Saint-Criq and Harvey, 2014; Sweezey and Ratjen, 2014). This observation is underlined by the UK Cystic Fibrosis Registry Annual Data Report 2019 by showing a 6 years lower median predicted survival for female CF patients compared to male CF patients when considering the years 2015-2019 (Cystic Fibrosis Trust, 2020). In spite of the high medical relevance for pathogenesis and patient treatment, there is still a lack of understanding the underlying mechanisms and key drivers (Silveyra et al., 2021). Notably, women on average develop chronic infections with mucoid *P. aeruginosa* 1.7 years earlier than men (Demko et al., 1995). Mucoid *P. aeruginosa* produces extraordinary large amounts of the polysaccharide alginate, and is known to be associated with frequent pulmonary exacerbations and resistance to the host’s immune defense and antibiotic treatments (Chotirmall et al., 2012).

Reasons for this sex disparity have not been fully clarified and are discussed controversially (Lam et al., 2021). They seem to be multifactorial, ranging from anatomic to nutrition status differences to hormonal differences between men and women as well as fluctuating sex hormonal serum levels affected by the season, day, age, weight, pregnancy, female cycle, the onset of menopause, or the use of hormonal contraceptives. In particular, the sex hormone 17β-estradiol ((17β)-estra-1,3,5(10)-triene-3,17-diol, hereinafter: E_2_) contributes to more unfavorable clinical prognosis for women with CF (Chotirmall et al., 2012; Saint-Criq and Harvey, 2014; Sweezey and Ratjen, 2014; Sutton et al., 2015).

The following modes of E_2_ action in CF disease progression are postulated: 1) an effect on calcium-controlled chloride ion channels (Imberti et al., 2018), 2) the modulation of the immune response during defense against infection (Abid et al., 2017), and 3) an E_2_ induced increase in specialized mucus-producing epithelial cells of the bronchial mucosa (Tam et al., 2014). From a microbiological point of view, there is evidence that the switch to a mucoid *P. aeruginosa* phenotype is also influenced by the patient’s blood serum E_2_ level (Chotirmall et al., 2012).

Very recently, it was shown that E_2_ can have a direct impact on *P. aeruginosa*, inducing bacterial motility, production of the virulence factor pyocyanin, as well as adhesion to and invasion into bronchoepithelial cells (Tyrrell and Harvey, 2020). Tyrrell et al. further investigated whether the addition of selective estrogen receptor modulators (SERMs) can reverse these E_2_ induced effects. The authors found that the mobility of *P. aeruginosa* in a semi-solid medium, meaning the swarming behavior, as well as the movement on surfaces promoted *via* type IV pili, meaning the twitching behavior, were significantly increased after adding E_2_ to the culture. The study resulted in a first hint that E_2_ modulates biofilm growth of the non-CF laboratory strain PAO1, but not for the two CF isolates from one patient they tested.

However, the underlining molecular mechanisms for such E_2_ induced physiological changes on bacterial pathogens like *P. aeruginosa* are still uncovered and looking at the bioavailability and metabolism of E_2_ by bacteria, there are many open questions. In 1992, it was first reported that *P. aeruginosa* expresses a cytosolic estrogen-binding protein with lower affinities also for other steroids (Rowland et al., 1992). Apart from that, it was reported that at least some environmental bacteria, including *P. aeruginosa*, are able to degrade and metabolize steroids for survival and proliferation, although it was observed that passive diffusion might be impeded by the lipopolysaccharide (LPS) leaflet on the outer surface of Gram-negative bacteria (Plésiat and Nikaido, 1992). Just recently, the degradation of E_2_ *via* 3-oxoacyl-(acyl-carrier protein) reductase (OAR) from *Pseudomonas citronellolisa* was reported (Fu et al., 2022). OARs have been also described in *P. aeruginosa* as an essential enzyme of the fatty acid biosynthetic pathway to produce QS signals and virulence factors like pyoverdine (Guo et al., 2019) that could be another link to how E_2_ can indirectly influence these relevant virulence processes just *via* its metabolism.

Until now, there is very limited knowledge on how E_2_ regulates biofilm development of *P. aeruginosa* in the context of CF and with respect to growth extent and its phenotype. Therefore, studies reporting on the direct impact of E_2_ on a broader collection of CF-*P. aeruginosa* isolates, which are known for their wide variation in phenotype and responsiveness to environmental conditions (Thöming et al., 2020), are needed.

Around 80% of microorganisms in the environment and in the body involved in infections, including *P. aeruginosa*, reside in biofilms (Jamal et al., 2018). They are attached to mucosal epithelia surfaces and built up by accumulated microorganisms that are encapsulated *via* a protective and highly hydrated mucous layer consisting of polysaccharides, proteins, and nucleic acids, and called the extracellular polymeric substance (EPS). The EPS facilitates optimal nutrient diffusion and protection against antibiotic treatments *via* biofilm-induced tolerance, recently proven for *P. aeruginosa* clinical isolates (Thöming and Häussler, 2022), or the survival of ‘persister cells’, a small subpopulation of non-dividing multi-drug resistant bacterial cells remaining after antibiotic treatment (Yin et al., 2019).

At the beginning of the biofilm’s life cycle a stronger irreversible attachment is established, e.g., mediated *via* type IV pili, which results in multilayered biofilm structures (Boudarel et al., 2018). During maturation, a spot-like aggregation of cells into microcolonies and a simultaneous production of EPS lead to matured biofilms with unique structures, which can be diverse amongst *P. aeruginosa*. Finally, a subpopulation of bacteria can actively leave the biofilm or passively detach due to shear forces to initiate a new colonization with subsequent formation of a new biofilm (Yin et al., 2019).

Microbial infections are controlled by a complex bidirectional pathogen-host crosstalk, involving inter-kingdom signaling molecules including hormones. QS systems mediate microbial communication and are essential for the regulation of the biofilm life cycle, including the formation of EPS and virulence factors. The link between the QS system and biofilm formation is best investigated for *P. aeruginosa* that uses acyl homoserine lactones (AHLs) (Babatunde et al., 2018; Mukherjee and Bassler, 2019). Recent studies found that host factors can also interfere with QS regulated biofilm growth (Blackledge et al., 2013). Biofilms are also known to adapt easily to environmental changes. Microbial infections are controlled by a complex bidirectional intra- and inter-kingdom crosstalk between such biofilm-forming pathogens and the host, which includes microbial signals like QS molecules and virulence factors from bacteria but also signaling molecules originating from the mammalian immune and hormonal system (Freestone, 2013).

*P. aeruginosa*, for instance, performs a series of adaptive processes during CF lung infections, which are influenced by specific factors in the host’s environment. This microbial adaptation mostly affects the biofilm phenotype or virulence, which in case of *P. aeruginosa* is reflected by the switch to mucoid phenotypes and an increased production of pyocyanin, both leading to a boost in pathogenicity and decline in lung function (Ryall et al., 2014; Thöming et al., 2020).

Considering the lack of knowledge in understanding how E_2_ impacts biofilm growth of CF- *P. aeruginosa* isolates, this study was devoted to performing a systematic investigation of *P. aeruginosa* isolates from several CF patients. Complementary approaches of biofilm analyses were used to get insights into the impact of the sex steroid hormone estradiol on biofilms of this CF lung pathogen. In doing so, we were able to show that estradiol not only enhances growth of biofilm but also induces ultrastructural changes in biofilms from a subset of CF isolates.

## Materials and Methods

### Source of Estradiol and Common Chemical Reagents, Media, and Buffer

17β-estradiol (here: E_2_), β-estradiol-water soluble (cyclodextrin-encapsulated, here: WSE_2_, 1 mg contains 48 mg E_2_, rest is 2-Hydroxypropyl-β-cyclodextrin), 2-Hydroxypropyl-β cyclodextrin (here: β-cyclodextrin), Dimethyl sulfoxide (DMSO) and crystal violet (CV) were purchased from Sigma-Aldrich (Steinheim, Germany). Ethanol (EtOH) was purchased from Carl Roth (Karlsruhe, Germany). LB (Lysogeny broth) was prepared by dissolving 7 g/l Sodium Chloride (Th Geyer), 5 g/l Yeast Extract (Carl Roth) and 10 g/l Tryptone/Peptone (granulated, Carl Roth) in distilled water.

Dulbecco’s phosphate buffered saline (DPBS, without calcium or magnesium; Lonza Walkersville, MD USA), buffers, Milli-Q water (Millipore Elix^®^ Advantage 3, Millipak^®^ Filter) and media were sterilized *via* autoclaving (121°C, 1.2 bar for 15 min) before usage.

### Non-CF *P. aeruginosa* Strains and CF *P. aeruginosa* Isolates

The bacteria included in this study and their origins are listed in **Supplementary Table S1**. *P. aeruginosa* isolates from CF patients have been described and analyzed in previous studies (Berchel et al., 2011; Le Gall et al., 2013; Mottais et al., 2018; Hornischer et al., 2019; Khaledi et al., 2020; Thöming and Häussler, 2022).

### Microtiter Plate Biofilm Model and E_2_ Treatment

*P. aeruginosa* glycerol stocks stored at -80°C were streaked onto Columbia agar with sheep blood plus (Oxoid Deutschland GmbH, Wesel, Germany). Overnight cultures were prepared by inoculating 3 ml of LB with a single colony and incubated at 180 rpm and 37°C (incubator MaxQ6000, Fisher Scientific, Hampton, NH, USA) for 17 h +/- 2 h.

**Figure1:**
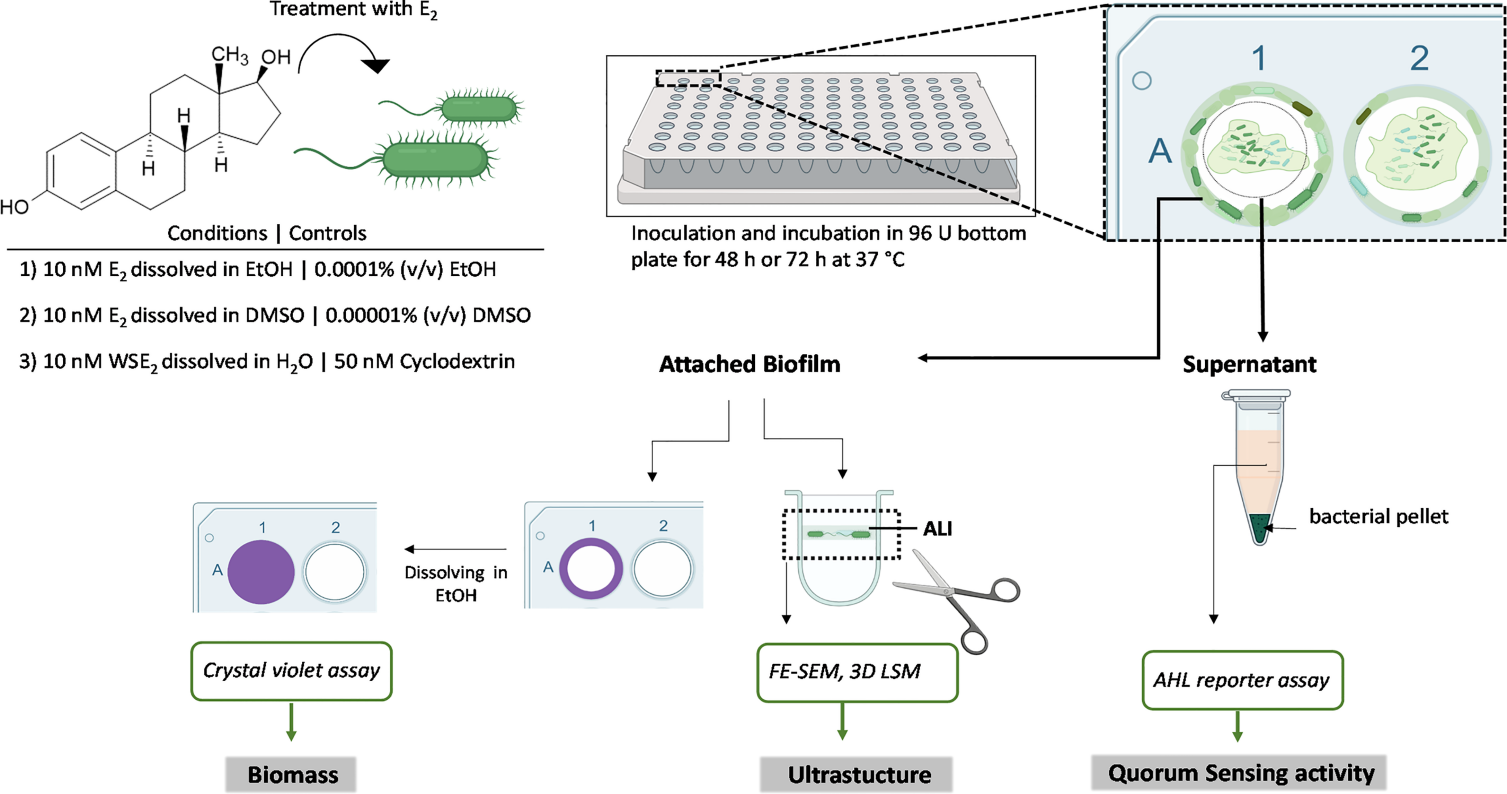
Integrated analysis of applied biofilm model. Biofilms of CF isolates were grown under static conditions on a PVC 96-well plate for 48 h or 72 h (in case of slow growing isolates) and both the attached biofilm mass as well as the QS activity in supernatants were analyzed. The attached biofilm was stained with crystal violet to quantify the biomass and sections of the PVC wells containing the biofilm at the air-liquid interface (ALI) were cut out and microscopically analyzed.

After adjusting the overnight culture in semi-micro cuvettes (1.6 ml Rotilabo single-use, Carl Roth) to an OD_600_ of 0.5 in LB, cultures were diluted 250-fold in LB. *P. aeruginosa* isolates were treated with E_2_ or EtOH, DMSO or β-cyclodextrin (solvent controls). E_2_ was dissolved in EtOH and DMSO, respectively while the WSE_2_ was dissolved in H_2_O with a final concentration of 10 nM (complexed with 50 nM β-cyclodextrin), if not indicated otherwise. Concentrations of EtOH and DMSO solvent controls were 0.0001% (v/v) and 0.00001% (v/v), respectively. Final concentration for β-cyclodextrin control was 50 nM. Biofilm formation was assessed in a Serocluster™ 96-well U bottom plate, polyvinyl chloride (PVC) (Corning^®^, Kennebunk, ME, USA) and sealed with an air-permeable membrane AeroSeals™ (Kisker Biotech GmbH & Co. KG). Isolates were placed in a humid stainless-steel chamber to avoid media evaporation and incubated for 48 h or, in case of slow growing isolates, C21-C2 and MHH0985 for 72 h at 37°C without shaking. After 24 h and additionally after 48 h for slow growing isolates 60 µL of medium containing solvent controls and E_2_ at the above indicated final concentrations was added to the microtiter plate according to an established biofilm model for susceptibility screenings (Thöming and Häussler, 2022) that was adjusted for this study. Biofilms were further analyzed as depicted in **Figure 1**.

### Crystal Violet Assay for Quantification of Adherent Biofilm

Non-adherent bacteria were washed off gently by placing the pipette tip in the middle of the well (biofilm mainly adhered to the walls of the wells), and washed thrice with 200 μl sterile H_2_O at 120 rpm for 5 min at 37°C (incubator MaxQ6000). Adherent bacterial biomass was determined *via* CV assay as previously described with slight adjustments (O’Toole, 2011). Briefly, biofilms were stained with 180 μl of 0.1% (v/v) CV dissolved in water and filtered to remove crystals. Plates were incubated for 30 min at room temperature (RT). The wells were washed again thrice with H_2_O while shaking them at 120 rpm for 5 min for each washing step at RT and the plates were tipped on tissues to remove excess stain. To dissolve CV bound to the biofilm 200 μl of 95% (v/v) EtOH was added to each well for 30 min at RT. Solutions were pipetted up and down thrice before transferring the solutions to a transparent flat bottom 96-well plate (Sarstedt AG & Co. KG, Nümbrecht, Germany) in order to measure the absorbance at OD_580_ with a multiplate reader (Tecan Infinite^®^ M200, Tecan Austria GmbH, Grödig/Salzburg, AUT).

Samples were diluted in a way, that final absorbence values are less than 1.0. For each treatment condition a minimum of six (up to eight) technical replicates (wells) were considered. Percentage of attached biofilm mass was calculated by subtracting the LB sterile control’s background from each sample. E_2_ induced changes of the percentage of attached biofilm mass was determined by dividing the absorbance at 580 nm as CV assay readout (after sterile LB background subtraction) of E_2_ treated biofilms by the absorbance of the corresponding solvent control and then multiplied with 100.

Experiments were repeated at least three times. Responsiveness measured *via* CV assay was defined as 10% change relative to control (> 110%, or <90%), for at least three independent experiments regulated in the same direction for an E_2_ treatment condition. Another requirement was that the arithmetic mean of all experiments had to exceed this threshold. CF isolates were defined to show no effect on a certain E_2_ treatment condition, if less than three independent experiments showed a regulation (more than 10%) in the same direction.

### FE-SEM Biofilm Sample Preparation and Analysis

PVC plates with biofilms were washed three times with PBS and fixed with 2.5% Glutaraldehyde/PBS over night at 4°C. After the plates were washed again three times with PBS, 200 µL of the following solutions were added to the plates and incubated at 20°C for 10 min for dehydration of samples: 50% EtOH, 70% EtOH, 80% EtOH, 90% EtOH. Afterward, plates were incubated with 100% EtOH for 10 min. Finally, 200 µL of hexamethyldisilazane (Sigma Aldrich, Steinheim, Germany) was added to each well for 30-60 s and plates were air dried before gold sputtering.

Sections of the well including the air-liquid interface (ALI) of the 96-well U bottom plates were cut out with a scalpel (first the bottom, then the upper part). A cut was then made on the side of each well to flatten it on a heated plate at 45°C by gently pressing it down with a tweezer on both sides. Samples were placed on a specimen stub containing carbon adhesive discs. The samples were sputter-coated with a thin gold layer (8 –10 nm) in a sputter coater (S150B, BOC Edwards, Bolton, UK) to increase the conductivity of the sample surfaces at a pressure of 0.2 mbar in an argon atmosphere for 2 min at a voltage of 1.0 kV.

The FE-SEM (Zeiss Ultra 55 cv, Oberkochen, Germany) measurements were performed with an operating voltage of 5.0 kV and the detector was the Inlens secondary electron detector. SmartSEM software was used for the collection and processing of the FE-SEM images. Documented areas of the ALI were defined to be positioned maximally 500 µm below the upper visible border of the biofilm.

Because the FE-SEM analysis lacks information about the Z-axis and therefore three-dimensional structure, the gold-sputtered biofilm samples were also analyzed *via* the 3D-laser scanning microscope LEXT OLS4000 (Olympus, Hamburg, Germany). Topographical images were measured based on the reflection from the surface of the biofilm samples. The data were processed and analyzed by using SPIP software (Image Metrology, Lyngby, Denmark) to create 3D pictures and determine the roughness parameter Sq. The tilt and curvature of the PVC plates was compensated by using 4th order flattening and measurement noise was removed by interpolation of individual pixel outliers. The topography is shown as 3D representation and the average area roughness Sq of the surface was evaluated in three areas (240x240 µm²) that are free of scratches/artefacts on the surface. The maximum roughness height Rt was determined *via* the Software Gwyddion 2.60 using linear interpolation and is based on 10 representative profiles over a minimal length of 0.25 mm and a maximal length of 0.7 mm.

### Quorum Sensing Reporter Assay

After growth of biofilms as described above supernatants were pooled from 6 wells and centrifuged at 26452 x g for 10 min and transferred to a new Eppendorf tube, sterile filtered (Rotilabo-syringe filter, PES, sterile, pore size 0.20 µm, Carl Roth) and kept on ice until measurement.

For the measurement of QS activity, that indicates the presence of AHLs in the biofilm supernatant, reporter strains *Escherichia coli* JM109 luxP LuxCDABE pSB401 and *Escherichia coli* JM109 luxP LuxCDABE pSB1075 (Winson et al., 1998) were used that detect different spectra of AHLs in the supernatant. AHLs N-Hexanoyl-DL-homoserine lactone (C6-HSL) and N-(3-Oxododecanoyl)-L-homoserine lactone (3-Oxo-C12-HSL) purchased from Sigma Aldrich were used as positive controls at a concentration of 10^-6^ M.

AHL reporter bacteria were overnight cultured by adding one colony to 5 ml LB containing a final concentration of either 10 µg/ml Tetracycline (pSB401) or 100 µg/ml Ampicillin (pSB1075) for 16 h at 200 rpm and 37°C. Afterwards, reporter bacteria suspensions were adjusted to an OD_600nm_ of 0.2 in LB.

Ninety µL of reporter bacteria *E. coli* pSB401 or *E. coli* pSB1075 were pipetted in triplicates into a white, sterile 96-well flat bottom microtiter plate (Greiner Bio-one, Frickenhausen, Germany). Thirty µL of supernatant, LB control, or LB containing 10^-6^ M synthetic AHLs were added to the well and sealed with transparent Ampliseal™ (Greiner Bio-One). Luminescence activity was measured *via* the multiplate reader (Tecan Infinite^®^ M200) for 20 h with a kinetic interval of 20 min at 30°C. The percentage of QS activity change upon E_2_ treatment was calculated based on the luminescence activity at timepoint 15 h (for pSB401) or 2 h (for pSB1075). After subtracting the luminescence background caused by the reporter strain for each measurement, mean resulting luminescence of the technical triplicates after E_2_ treatment was divided by the corresponding luminescence of the mean control condition and multiplied with 100.

### Data Processing and Statistical Analysis

Standard deviations were calculated *via* Excel (STDEV.P) and further calculations with mean values were performed according to standard deviation propagation. Statistical analyses of CV assay data and QS reporter assays were performed using GraphPad Prism to compare E_2_ induced changes (in terms of attached biofilm mass or QS activity in %) with control condition for a minimum of three independent experiments. One sample t-test compared to a theoretical mean of 100% was performed for each condition. Significant induction or reduction of attached biofilm was defined as p ≤ 0.05. Non-significant response (here induction) means that 10% more attached biofilm mass was measured relative to control for at least three independent experiments as well as the arithmetic mean of all performed experiments. The remaining CF isolates with effects lower than this 10% were dedicated as “no effect”.

## Results

The effect of E_2_ on biofilm development of CF isolates was tested as shown in the experimental setup in **Figure 1**. Because E_2_ is barely water soluble we used EtOH for comparison with studies of others (Tyrrell and Harvey, 2020), and DMSO as solvents to carry out experiments under aqueous conditions. As a third condition we used a water soluble E_2_ that contains 50 nM β-cyclodextrin for complexation of E_2_ allowing its solubility in H_2_O to obtain a final concentration of 10 nM E_2_. All these treatment conditions were compared to the corresponding solvent controls in order to normalize result to possible solvent effects on bacteria. Our integrative approach allows to perform several readouts, including biofilm mass quantification *via* CV assay, characterization of ultrastructure *via* FE-SEM, height profile, and roughness *via* 3D LSM on the same biofilm substrate. Additionally, QS activity of biofilm culture supernatants was determined *via* AHL reporter assays (**Figure 1**).

### CF Isolates Respond to E_2_ With Increased Attachment of Biofilm Mass *In Vitro*

**Figure 2:**
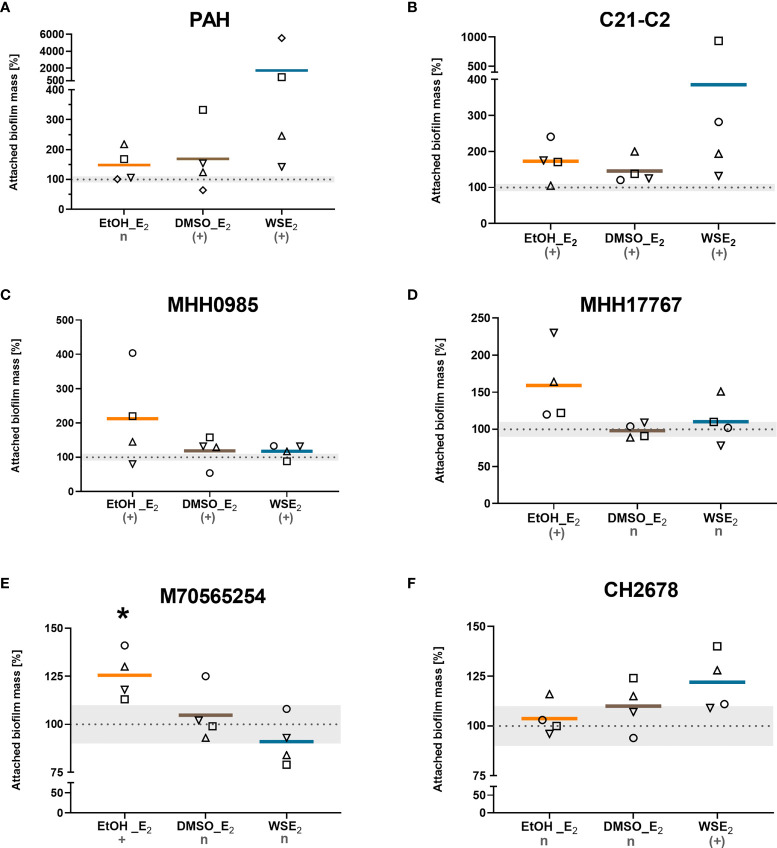
CF isolates that showed E_2_ induced attached biofilm mass. CF isolates PAH **(A)**, C21-C2 **(B)**, MHH0985 **(C)**, MHH17767 **(D)**, M70565254 **(E)**, and CH2678 **(F)** were treated either with 10 nM E_2_ in EtOH (orange), E_2_ in DMSO (brown), WSE_2_ (blue), or corresponding solvent controls (see **Figure 1**) for 48 h or 72 h in case of **(B, C)**. Attached biofilm mass was quantified *via* CV assay. Absorbance of the corresponding solvent control was set to 100% (see dotted line). Colored horizontal lines indicate mean values of four independent experiments (◯ = experiment 1, ⬜ = experiment 2, △ = experiment 3, ▽ = experiment 4). E_2_ responsiveness was defined as more than 10% change (indicated by the grey zone between 90% and 110%). *: p ≤ 0.05 (n=4). n: no effect, (+): induction, +: significantly induced (see *Materials/Methods* for detailed description).

From the 10 investigated CF isolates, six were found responsive to E_2_ at least for one of the tested E_2_ treatment in at least three of four performed independent experiments (**Figure 2**).

Two isolates (C21-C2 and MHH0985) showed E_2_ induced biofilm attachment for all three conditions tested that use different approaches to bring E_2_ in solution (EtOH, DMSO, and β-cyclodextrin in water).

Further, we identified a PAH increase in attached biofilm for both E_2_ in DMSO and WSE_2_. MHH17767 and M70565254 responded with an increase in attached biofilm mass to E_2_ in EtOH with the latter exhibiting a significant effect compared to control, while CH2678 only responded to WSE_2_. Treatment with WSE_2_ resulted in the strongest mean attached biofilm mass induction of more than 17-fold for PAH (**Figure 2A**), followed by treatment with E_2_ in EtOH, exceeding a mean increase of around 2-fold for isolate MHH0985 (**Figure 2C**).

To investigate if the induction of attached biofilm mass is indirectly caused by an overall enhanced planktonic growth kinetics, four of the E_2_ responsive *P. aeruginosa* isolates were analyzed under the six different growth conditions for 24 h (E_2_ treatment and corresponding solvent control). Growth dynamics did not result in any relevant changes in doubling times or maximal endpoint OD_600 nm_ upon E_2_ treatment for any of the tested CF isolates (**Supplementary Figures S4A, B**). There was also no concentration dependent effect on planktonic growth detectable for WSE_2_ concentrations ranging from 10^-9^ to 10^-6^ M (**Supplementary Figure S4C**).

**Supplementary Figure S5** illustrates that four other CF isolates (C4-C1, MHH15204, MHH16563, and MHH2419) were considered non-E_2_ responsive to any of the E_2_ treatment conditions according to the above-mentioned criteria.

To answer the question if E_2_ causes the same effect on broadly used non-CF *P. aeruginosa* strains in our biofilm model that are isolated from other human tissue than the respiratory tract, we tested the non-CF strains ATCC19660 and PAO1.

**Figure 3:**
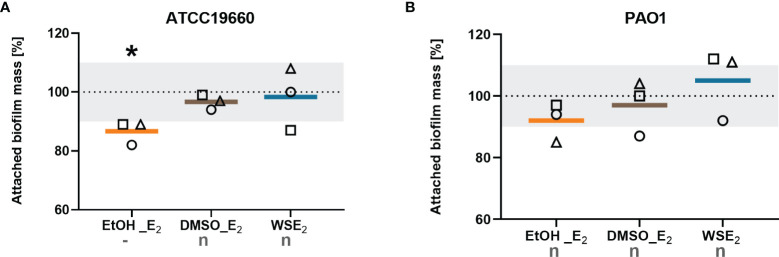
Non-CF strains did not show increase in attached biofilm mass upon any E_2_ treatment. *P. aeruginosa* strains ATCC19660 **(A)** and PAO1 **(B)** were treated with either 10 nM E_2_ in EtOH (orange), E_2_ in DMSO (brown), WSE_2_ (blue), or corresponding solvent controls for 48 h (see **Figure 1**). Attached biofilm mass was quantified *via* CV assay. Solvent controls were set to 100% (see dotted line). Colored horizontal lines indicate mean values of three independent experiments (◯ = experiment 1, ⬜ = experiment 2, △ = experiment 3) with each single experiment based on at least six technical replicates. E_2_ responsiveness was defined as more than 10% change (indicated by the grey zone between 90% and 110%). *: p ≤ 0.05 (n=3). n: no effect, -: significantly reduced (see *Materials/Methods* for detailed description).

Non-CF strains did not show any E_2_ responses for most of the tested conditions. Only the treatment of *P. aeruginosa* ATCC 19660 with E_2_ in EtOH resulted in a significant decrease in biofilm attachment that means a contrary effect compared to E_2_ responsive CF isolates. PAO1 turned out to be non-responsive toward E_2_ regarding a change in attached biofilm mass (**Figure 3**).

**Table 1** summarizes the E_2_ effects determined for every strain in the various conditions studied, thus distinguishing between E_2_ responsive that react with induction or reduction of attached biofilm mass to at least one E_2_ treatment condition, and E_2_ non-responsive strains according to the criteria considered.

**Table 1:** Overview of E_2_ effects on biofilms from CF and non-CF isolates.

Tested *P. aeruginosa*		EtOH_E_2_	DMSO_E_2_		WSE_2_
**Non-CF strains**					
1	PAO1	n		n	n
2	ATCC 19660	-		n	n
**E_2_ responsive CF isolates**					
1	C21-C2	(+)		(+)	(+)
2	MHH0985	(+)		(+)	(+)
3	PAH	n		(+)	(+)
4	MHH17767	(+)		n	n
5	M70565254	+		n	n
6	CH2678	n		n	(+)
**Non-E_2_ responsive CF isolates**					
1	C4-C1	n		n	n
2	MHH15204	n		n	n
3	MHH16563	n		n	n
4	MHH2419	n		n	n

**+**: attached biomass was significantly induced (p < 0.05) **(+)**: at least three independent experiments; showed E_2_ induced attached biofilm mass; **-**: attached biomass was significantly reduced (p < 0.05). **n**, no effect.

In summary, our data show that E_2_ does specifically enhance growth of attached biofilms for more than half of the tested CF isolates, which is independent from planktonic growth behavior. Such E_2_ effect was not observed with the non-CF strains PAO1 and ATCC19660.

### E_2_ Induces Changes in Biofilm Structure of E_2_ Responsive CF Isolates

**Figure 4:**
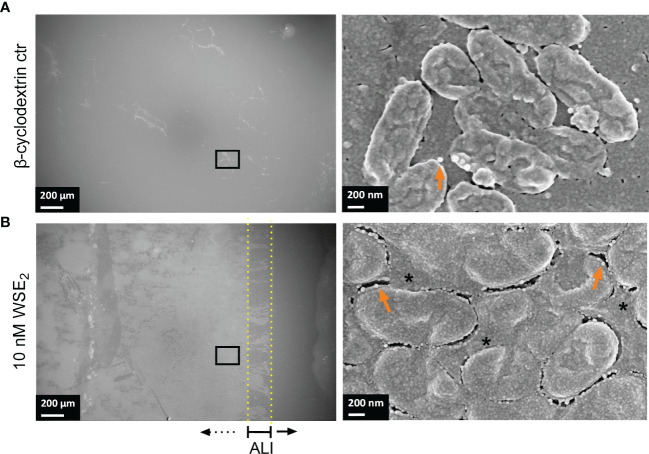
WSE_2_ strongly induces growth of attached PAH biofilm. Overview of exemplary PAH biofilms grown in 50 nM β-cyclodextrin (CD) as solvent controls **(A)** or 10 nM WSE_2_ **(B)**. Biofilm samples were cut out from PVC well plates and analyzed *via* FE-SEM. Black rectangle selection indicates area chosen for further magnification to visualize single bacteria (right). ALI: Air-liquid-interface (yellow dotted line, not visible for A). Orange arrows: outer membrane vesicles; *: compact EPS. Scale bar: 200 µm (left), 200 nm (right). Additional measurements of the sample: attached biofilm mass (**Figure 2A**, experiment No. 4◊), biofilm surface coverage (**Supplementary Figure S6C**) and height profile/roughness (**Figure 7**).

**Figure 5:**
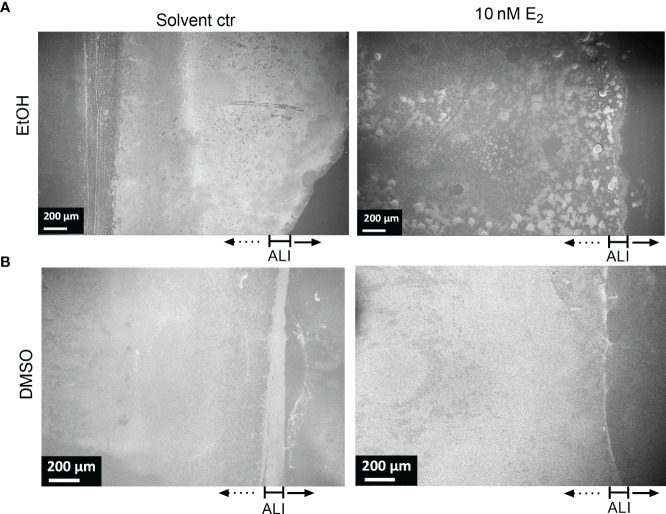
Structure of PAH biofilms treated with E_2_ dissolved in EtOH and DMSO. Overview of exemplary PAH biofilms treated with either 10 nM E_2_ in EtOH (**A**, right) or in DMSO (**B**, right) in comparison to the corresponding solvent controls (left). Biofilm samples were cut out from PVC well plates and analyzed *via* FE-SEM. ALI, Air-liquid interface. Scale bar: 200 µm. Additional measurements of the sample: attached biofilm mass (**Figure 2A**, experiment No. 4◊), biofilm surface coverage (**Supplementary Figure S6**) and height profile/roughness (**Figure 7**).

The quality of the biofilm was also investigated microscopically with a focus on surface coverage, surface ultrastructure, height profile, and roughness. We analyzed those CF isolates more in detail that responded to E_2_ based on the CV assay (PAH, C21-C2, and MHH17767, **Figure 2**) as well as PAO1 as lab strain that did not show any change in attached biofilm mass (see **Figure 3**). The strongest effect on ultrastructure upon E_2_ treatment was observed for the isolate PAH already on a low magnified overview of the overall biofilm for all used solvents showing the extent of biofilm formation and the arrangement of microcolonies (**Figures 4**, **5**). β-cyclodextrin alone had an inhibitory effect on biofilm attachment compared to the other solvent controls. WSE_2_ could recover biofilm attachment, as shown in **Figure 4** and as determined by CV assay indicating on average a more than 17-fold higher attached biofilm mass (**Figure 2A**). In addition, quantification of surface coverage based on low magnified SEM pictures showed seven times higher biofilm surface coverage compared to β-cyclodextrin control (**Supplementary Figure S6C**).

The most obvious structural difference of the overall PAH biofilm was observed for E_2_ in EtOH. Throughout the overall biofilm, but especially at the air-liquid interface, a structural difference toward prominent microcolonies were observed compared to the more homogeneously grown biofilm under solvent control conditions (**Figure 5**).

Also, such a tendency of bacterial agglomeration into microcolonies embedded in a complex network of EPS could be detected for PAH biofilms grown on transwell insert substrates under the influence of E_2_ dissolved in EtOH (**Supplementary Figure S7**). Treatment with E_2_ dissolved in DMSO resulted in a different distribution of the overall biomass. For the DMSO control biofilm density was higher at the air-liquid interface (**Figure 5B**, left) while the E_2_ treated biofilm seemed to build up a dense biofilm including submerged areas of the well substrate (**Figure 5B**, right). Similar observations were reflected by isolate MHH17767 that was also accompanied by the appearance of elongated cells (**Supplementary Figure S10**). This influence of E_2_ dissolved in DMSO on the overall biofilm growth is underpinned by a slight increase of the biofilm-covered area based on the image analysis of SEM pictures, where E_2_ in DMSO induced a higher coverage area compared to control condition for PAH and C21-C2 (**Supplementary Figure S6**).

**Figure 6:**
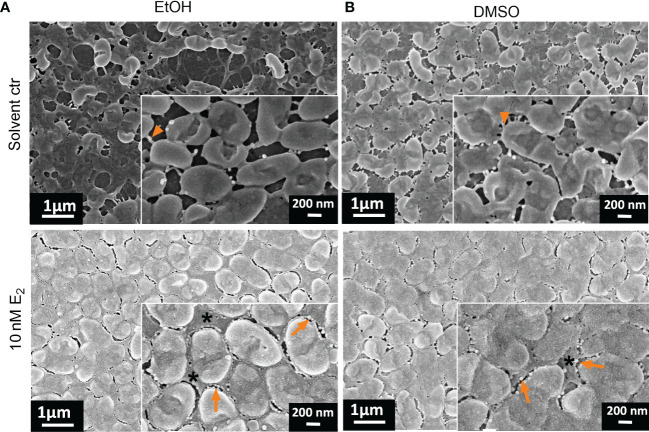
Ultrastructural changes of PAH biofilms treated with E_2_ dissolved in EtOH or DMSO. Overview of exemplary PAH biofilms treated with either 10 nM E_2_ in EtOH (**A**, down) or in DMSO (**B**, down) in comparison to the corresponding solvent controls [**(A**, **B)** up]. Biofilm samples were cut out and analyzed *via* FE-SEM. Arrowheads: visible bacterial cell-cell connecting appendages; arrows: outer membrane vesicles; *: compact EPS; scale bar: 200 µm; scale bar inserts: 200 nm. Additional measurements of the sample: biofilm mass (**Figure 2A**, experiment No. 4◊), biofilm surface coverage (**Supplementary Figure S6**) and height profile/roughness (**Figure 7**).

The highest magnification with single bacteria resolution illustrates the surface pattern of bacteria and differences in the EPS covering the bacteria and filling the extracellular gaps between bacteria (**Figures 4**, **6**). This can be clearly differentiated between the WSE_2_ and the β-cyclodextrin control condition for PAH biofilms. For the control condition only single aggregates of few bacteria disconnected *via* EPS are visible, while bacteria within the dense biofilm at the air-liquid interface are embedded in a homogeneously distributed EPS (**Figure 4**, right) similar to the PAH biofilms treated with E_2_ dissolved in EtOH or DMSO (**Figure 6**, lower row).

This EPS is so condensed that visible defined extracellular bacterial cell surface appendages probably reflecting pili and cup fimbriae or amyloid fibrils under solvent control conditions (**Figure 6**, arrowhead in magnified inserts) are not visible anymore after E_2_ treatment, where the intercellular space between the bacteria seemed to be homogenously filled with EPS embedding the bacteria.

Apart from PAH we also analyzed the E_2_ responsive isolate C21-C2, where FE-SEM analysis did not reveal any ultrastructural changes aside a slight increase of bacterial extracellular appendages that were more visible after treatment with E_2_ dissolved in DMSO compared to DMSO control (**Supplementary Figure S8B**). However, the lab strain PAO1 that already exhibited a strong biofilm forming phenotype under control conditions, showed some slight structural changes upon E_2_ treatment (**Supplementary Figure S9**) although attached biofilm mass was unchanged (**Figure 3B**). Especially, under treatment with E_2_ in EtOH and WSE_2_ bacterial appendages mediating cell-cell connections seemed to degenerate and form more extracellular vesicles compared to control.

**Figure 7:**
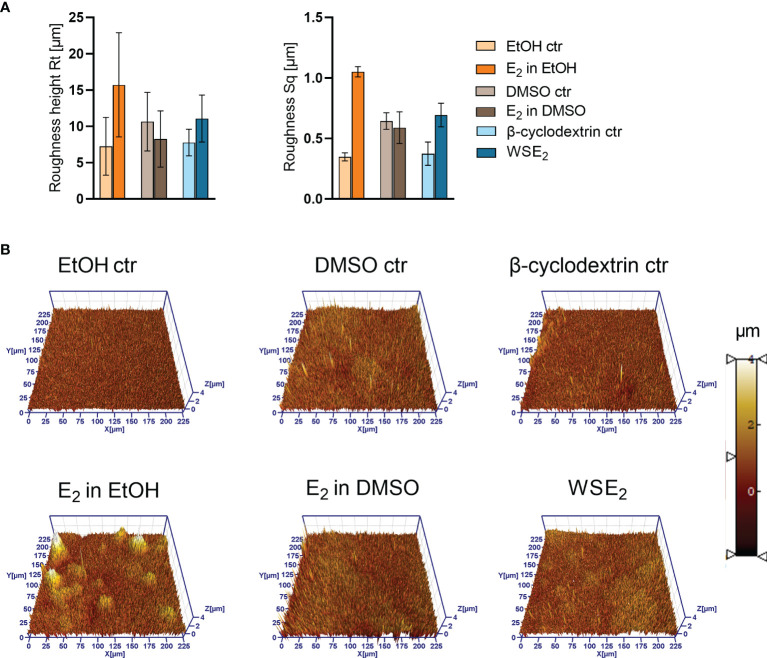
Height profile and roughness of PAH biofilms is changing upon E_2_ treatment as measured *via* 3D LSM. Same samples have been analyzed *via* FE-SEM as indicated in **Figures 5**, **6**. A Roughness height Rt (**A**, left) and RMS (root mean square average roughness Sq) (**A**, right) have been determined based on the three 3D LSM pictures (example shown in **B**). Compare overview pictures in **Supplementary Figure S11**).

Next, we analyzed the structure of PAH biofilms with the same gold-sputtered samples using 3D LSM to gain insights in the 3D structure, height profile and roughness, which confirmed the E_2_ induced changes in biofilm structure. Considering the height profile, it turned out that E_2_ dissolved in EtOH induced the formation of prominent microcolonies with a height of around 10 µm, indicating that these colonies contain several layers of bacteria (**Figure 7**).

The roughness measurement revealed an increase in roughness for PAH biofilms treated with E_2_ in EtOH or WSE_2_, indicating a rearrangement of the biofilm architecture. However, when considering the resolution at the applied magnification on level of bacterial aggregates, this finding may also indicate a remodeling of the EPS.

### E_2_ modulates Quorum Sensing Activity in E_2_ Responsive CF Isolates

Since QS plays a major role in the control of biofilm formation (Brindhadevi et al., 2020), we further collected supernatants from *P. aeruginosa* biofilms treated with E_2_ or the corresponding solvent control. QS activity was evaluated using the bioluminescence reporter strains *E. coli* pSB401 and *E. coli* pSB1075 as AHL biosensors (Winson et al., 1998), respectively, that have different sensitivity and responsiveness to long chain and short chain AHLs as illustrated in the **Supplementary Figure S12**. QS activity of supernatants from non-treated control biofilms of CF isolates PAH and C21-C2 was indicated by both reporter bacteria with C21-C2 supernatant resulting in a 60 times stronger luminescence signal for the pSB401 reporter strain than with PAH, which might be a hint to a higher concentration of short length AHLs (**Supplementary Figure S12D**).

**Figure 8:**
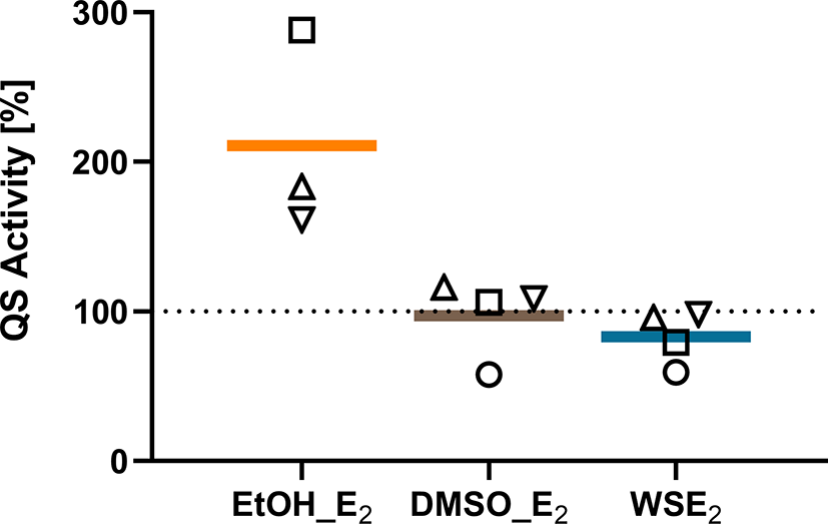
QS activity of C21-C2 biofilm supernatants measured by the luminescence reporter bacterium *E. coli* pSB401 is induced by E_2_ in EtOH. QS activity of supernatants from C21-C2 biofilms treated with 10 nM E_2_ in EtOH (orange), E_2_ in DMSO (brown), or WSE_2_ (blue) is compared relative to corresponding solvent control, which was set to 100% (dotted line). Each symbol represents an independent performed biofilm experiment with supernatants collection as biological replicate (○ = experiment 1, □ = experiment 2, ▵ = experiment 3). QS reporter assays have been performed for each supernatant and condition in technical triplicates.

Due to the prominent QS activity of C21-C2 biofilms measured by the pSB401 reporter bacteria, we investigated supernatants of biofilms treated with solvent control or E_2_ and found a prominent upregulation of QS activity on average two times higher for E_2_ in EtOH compared to EtOH control. In comparison WSE_2_ rather tends to reduce QS activity compared to β-cyclodextrin (**Figure 8**).

Similar but less prominent effects on QS activity could be measured with the pSB1075 for C21-C2. However, PAH seems to be less responsive to QS activity changes, and QS regulation was just detected for WSE_2_ with the reporter pSB401 (**Supplementary Figure S13**).

## Discussion

Microbial endocrinology is an emerging field relevant for understanding the impact of host environmental factors like hormones and how they influence infection progression on host mucosal surfaces (Neuman et al., 2015; Vemuri et al., 2019). With the recent insights into sex differences regarding the outcome of chronical lung diseases, specifically CF (Lam et al., 2021), sex steroid hormones moved into the spotlight of this research field (Silveyra et al., 2021). In the present work, the *in vitro* effects of E_2_ on *P. aeruginosa* biofilms were investigated on a panel of 10 clinical isolates each coming from a different CF patient (**Supplementary Table S1**).

We herein report a series of data characterizing possible effects toward *P. aeruginosa* biofilm growth. Considering the attached biofilm mass, the herewith investigated CF isolates can be divided into E_2_ responders or non-E_2_ responders. We found in the applied biofilm model that the attached biofilm mass is increased *via* E_2_ for six out of 10 analyzed CF isolates (**Figure 2**, **Table 1**, and **Supplementary Figure S5**). It turned out that the extent of this biofilm inducing effect is underlying high variations among the clinical isolates, which exhibits diverse phenotypes and growth characteristics on blood agar plates (see **Supplementary Table S2**; **Supplementary Figures S1-2**) but also differences in biofilm ultrastructure indicated *via* FE-SEM and 3D LSM analysis of this study (**Figures 4**-**7**).

Three E_2_ responsive CF isolates that showed an increase in biofilm mass (PAH, C21-C2, and MHH17767) were microscopically analyzed *via* FE-SEM. Strong structural differences between E_2_ treatment and corresponding control have been observed especially for PAH, therefore, these biofilms were analyzed additionally *via* 3D LSM to measure height profiles and surface roughness. WSE_2_ treatment condition revealed the strongest E_2_ effect indicated not only *via* CV assay results with over 17 times more attached biofilm mass (**Figure 2A**, experiment No. 4◊) but also seven times higher surface coverage (**Supplementary Figure S6C**). Thus, E_2_ could recover biofilm formation compared to the β-cyclodextrin control (**Figure 4**).

For EtOH a structural change of PAH biofilms toward growth into microcolonies was observed, which reduced homogeneity of the biofilm and enhanced microcolony thickness as well as surface roughness (**Figure 7**). Simultaneously, the number of bacteria between these microcolonies was reduced (**Figure 5A**). This was confirmed also on transwell insert substrates, which mediate slightly different biofilm growth conditions than PVC plates (**Supplementary Figure S7**). The visible E_2_ triggered change in morphotype might result from an early process during the initial steps of biofilm development. In other studies, it was reported that E_2_ influences the mobility of *P. aeruginosa* in a semi-solid medium, meaning the swarming behavior, as well as the movement on surfaces, called twitching behavior (Tyrrell and Harvey, 2020). These mobility modes are also determining biofilm phenotype and involve type IV pili, which can repetitively extend and retract to promote movements (Otton et al., 2017). A modulation of swarming or twitching behavior may therefore result in a migration of bacteria toward microcolonies (Rossi et al., 2018).

Other bacterial appendages that are not certainly distinguishable *via* FE-SEM from type IV pili are curli fibers (fab-based amyloid fibrils), which can enhance biofilms mechanically and protect them from desiccation (Erskine et al., 2018). Such bacterial appendages could be clearly seen under solvent control conditions for PAH in the intercellular space. After E_2_ treatment they disappeared due to degeneration or abundant filling of the intercellular space with EPS (**Figure 6** and **Supplementary Figure S7**), which is likely composed of *Pseudomonas* specific exopolysaccharides such as Pel, Psl, and alginate or DNA (Wei and Ma, 2013).

According to Chotirmall et al., E_2_ can induce alginate production in *P. aeruginosa* after prolonged exposure *in vitro.* Based on the Irish Cystic Fibrosis Registry this study further revealed that *P. aeruginosa* found in CF patients undergoes two times more frequent mucoid conversion in female than male patients (Chotirmall et al., 2012).

This observation could be an explanation for the observed homogenous extracellular filling of PAH biofilm with EPS found in our study, although CF isolates were exposed for shorter time here.

Due to the complex composition of EPS such a hypothesis of EPS remodeling has to be further proven biochemically. In this regard, one needs to emphasize that an overproduction of alginate cannot be considered responsible for the increase of attached biofilm mass for all E_2_ responsive CF isolates investigated in this study because polysaccharide production can influence biofilm development but is dispensable for biofilm formation (Stapper et al., 2004).

Similar effects regarding the reduction of bacterial surface appendages after E_2_ treatment could be observed for the strain PAO1 although attached biofilm mass of this strain did not change (**Figure 3**) and PAO1 showed for all conditions a thick biofilm with homogeneous surface coverage (**Supplementary Figure S9**). The FE-SEM analysis revealed specific changes of the outer membrane/extracellular space triggered by E_2_. Bacterial surface appendages like pili or curli fibers seemed to be reduced for E_2_ in EtOH or WSE_2_ while outer membrane vesicles (OMV) with a diameter of around 50 nm was prevalent, a size in line with previous reports (Mozaheb and Mingeot-Leclercq, 2020). OMVs have been also visible for PAH and after E_2_ treatment (**Figures 4**, **6**). Such OMV can indicate stress and mediate transfer of resistance or virulence factors as well as dynamic structural outer membrane changes.

When comparing the morphology changes of different CF isolates, we obtained heterogeneous results. Isolate MHH17767 exhibited a remarkable compact homogenous biofilm structure with no obvious changes upon E_2_ treatment apart from cell elongation that could be only observed for E_2_ in DMSO treatment condition (**Supplementary Figure S10B**). Also, we observed elongated cells with the EtOH solvent control condition for C21-C2, which were not present after treatment with E_2_ in EtOH (**Supplementary Figure S8A**). According to previous reports, cell elongation of *P. aeruginosa* seems to be a morphological change due to missing cell division under anaerobic growth, a strategy the pathogen uses to adapt to thickened mucus layers, as found for instance in CF patient airways (Yoon et al., 2011).

Our results can be compared with previous data from the literature. First, our observations contrast with the findings by Tyrrell et al. reporting no effect of E_2_ on biofilm growth of CF isolates (Tyrrell and Harvey, 2020). However, it is worth mentioning that (i) this previous study focused on two CF isolates collected from a single patient at different time of the disease progression and (ii) some CF isolates were also identified as non-E_2_ responders in our study (**Supplementary Figure S5**). Beside this, we did not find any induction of biofilm attachment for the reference strains PAO1 and ATCC19660 (**Figure 3**) as reported before (Tyrrell and Harvey, 2020). Such differences cannot only be caused by a slight difference in biofilm models and growth conditions but also deviating E_2_ treatment conditions, starting from the concentration of stock solution and corresponding final EtOH concentration in this case, what they used as the only condition to solve E_2_. Also, the different character of ATCC19660 with septicemia from a very different host environment may be one explanation for the opposite biofilm reducing response to E_2_ (Schook et al., 1976).

The mechanisms that are transducing the E_2_ effect are still not clarified. Therefore, we further tried to gain insights by investigating the planktonic growth as a driving force but also QS as a switching mechanism. The slight increase in total planktonic growth for PAH or the slightly reduced doubling time of MHH17767 (**Supplementary Figures S4A, B**) cannot alone explain the observed strong increase in attached biofilm mass (**Figure 2**). Building up on existing knowledge about the impact of the QS system on mucoid switch but also on biofilm formation dynamics of *P. aeruginosa* (Tahrioui et al., 2019; Brindhadevi et al., 2020), we performed QS assays indicating a different extent of QS activity amongst the tested CF isolates PAH and C21-C2 (**Supplementary Figure S12D**). Moreover, we obtained first indications for an E_2_ triggered mechanism *via* the QS system for CF isolate C21-C2, which showed increased QS activity after treatment with 10 nM E_2_ in EtOH (**Figure 8**). In contrast, another group showed that supraphysiological concentrations of E_2_ (367 µM), which are 3.7 x 10^4 times higher than the concentration used in this study, can inhibit the QS system of the non-CF lab strain PAO1 *via* binding to the LasR receptor and thereby interfering with QS (Beury-Cirou et al., 2013). This suggests that non-physiological, supraphysiological concentrations compared to the here used concentration (10 nM), which is closer to physiological E_2_ blood serum concentrations (0.1-1.6 nM) (Chotirmall et al., 2012), can result in very different outcomes. Apart from the E_2_ concentration aspect, differences in basal QS activities between the highly variable CF isolate and the non-CF strains PAO1 (**Supplementary Table S1**) used in this previous study may add to the observed discrepancy.

Due to the high variability of E_2_ response of CF isolates, it still has to be investigated in depth, which genetic background responsive CF isolates have in common, and which preconditioned morphotype is most susceptible for E_2_. Especially the switch from low adhesive to strong biofilm forming bacteria as well as the remodeling of the EPS that we could observe *via* FE-SEM needs to be uncovered in future studies. Also, the detailed mechanism of how E_2_ interacts with *P. aeruginosa*, whether it is directly mediated *via* an estradiol binding receptor, indirect effects of central signaling pathways (QS, c-GMP, etc.), or side products of the E_2_ metabolism are involved, is still an open question. Beside this, the high polymorphism reported in this study may imply that the basic starting condition and phenotype of the CF isolates are critical with respect to the effect of E_2_ on biofilm growth. These variations are not surprising, because phenotypes are known to be highly polymorphic amongst CF isolates and non-CF strains like PAO1 (Mowat et al., 2011; Thöming et al., 2020; Camus et al., 2021; Jurado-Martín et al., 2021). Especially for CF, it is known that *P. aeruginosa* undergoes evolutionary processes in the course of an infection and can adapt its phenotype to the host’s environment (Bragonzi et al., 2009; Fischer et al., 2021).

Strikingly, all six E_2_ responsive CF isolates were resistant toward at least two out of five antibiotic classes, PAH and C21-C2, two highly responsive CF isolates, that have been investigated in more detail in this study, lack susceptibility toward four out of five antibiotics (**Supplementary Table S2**). Due to the high prevalence of multi-drug resistant *P. aeruginosa* in the CF lung, more data are needed to prove if there is a correlation between E_2_ responsiveness and multi-drug resistance. Also, a small colony variants (SCVs) phenotype (Häußler et al., 1999) could precondition the impact of E_2_ on biofilm growth, when we consider that most of the E_2_ responsive CF isolates show a small colony morphotype

Finally, some methodical limitations of this study need to be mentioned. First, floating *P. aeruginosa* aggregates in the biofilm supernatant, which are physiologically relevant in CF regarding susceptibility toward antibiotics (Jennings et al., 2021), have not yet been investigated. Second, the solvent effect may have a strong impact and our results emphasize the relevance of the chosen condition how E_2_ is dissolved in *in vitro* biofilm models. Variations between the effect of different treatment conditions may be partly caused by possible side effects of the solvent used. Although the lowest solvent concentrations possible have been used in this work, there are certain reported effects EtOH (Lewis et al., 2019), DMSO (Singh et al., 2021), and β-cyclodextrin (Ikeda et al., 2002; Oishi et al., 2008) possibly have. Third, the here used E_2_ concentration was chosen for comparability with previous *in vitro* studies (Chotirmall et al., 2012; Tyrrell and Harvey, 2020) and was around 10 times higher than reported physiological concentrations in CF-sputum (Chotirmall et al., 2012), which need to be addressed by concentration gradients.

Although showing some relevance, it is obvious that the *in vitro* model used in this study is very simplified compared with *in vivo* settings in CF lung airways. It is noteworthy that this study rather considered the endpoint of the biofilm life cycle. Further complementary analysis of different biofilm formation stages could provide additional insights into the E_2_ action mode. Considering host parameters, this model may be further implemented (e.g., with adjacent lung epithelial cells, immune cells, patient mucus, and resident lung microbiota) in order to reproduce bidirectional pathogen/host crosstalk, involving inter-kingdom signaling molecules including hormones. It is likely that ‘host imprinting effects’ (i.e., the *in vitro* persistence of bacteria responsiveness to E_2_ due to *in vivo* specific conditioning host parameters) can also contribute to the variations observed. Broadly speaking, *in vitro* microbiology should be further accompanied by clinical studies about the medical relevance of the microbial endocrine effects of E_2_ on *P. aeruginosa* biofilms, E_2_ concentrations in the lung, and thereby confirm the relevance of new insights of a direct E_2_ effect on *P. aeruginosa* biofilm on the outcome of *P. aeruginosa* infection in CF. Chotirmall et al. did an extensive statistical analysis of the Irish CF patient registry showing that patients taking hormonal contraceptives had strikingly fewer pulmonary exacerbations and required less antibiotic treatment (Chotirmall et al., 2012). A complete understanding of the impact of the patient’s hormonal status and application of hormonal contraceptives on lung pathogens like *P. aeruginosa* could lead to a final clear advice regarding hormonal medications of CF patients that can make a game-changing difference in disease outcome. However, this study adds to few other studies that explicitly focused on the impact of E_2_ on biofilm formation. In the context of other bacterial pathogens like uropathogenic *Escherichia coli*, it was shown that biofilm formation and the overall virulence trait was increased when applying 5 pg/ml E_2_ (Engelsöy et al., 2021).

In summary, this study further emphasizes the complexity of the multifaceted hormonal E_2_ effects on *P. aeruginosa* biofilm growth, which is likely to be better addressed thanks to an integrated approach as that used in the present work. From this point of view, this study should be considered as a starting point for further investigation of the underlying mechanisms and qualitative changes of E_2_ modulated clinically relevant *P. aeruginosa* biofilms. Our findings provide one further tentative explanation for the sexual dimorphism in bacterial infections associated with biofilm formation in CF and other chronical respiratory diseases associated with *P. aeruginosa* lung infections.

## Data Availability Statement

The original data supporting the findings of this study are included in the article/**Supplementary Material**. Further information can be provided by the corresponding author.

## Author Contributions

JA: investigation (most of experimental work), methodology, data analysis and curation, manuscript drafting, final revision. FA: methodology, investigation, data analysis (SEM and growth curve analysis). AA: investigation, data analysis (FE-SEM). DN: investigation (SEM sample preparation), data analysis; AK: investigation (3D LSM), DW: data analysis and curation (3D LSM). TM: funding acquisition and manuscript revision. TLG: methodology, data interpretation, manuscript writing-revision and editing and funding acquisition. MM: Conceptualization, data interpretation, manuscript drafting/final revision and funding acquisition. All authors gave their specific input to the manuscript according to their contribution and finally approved the manuscript.

## Funding

Mukoviszidose Institut gGmbH, Bonn, the research and development arm of the German Cystic Fibrosis Association Mukoviszidose e.V., the Christiane Herzog foundation; the University of Siegen (in particular the Open Access Publishing Fund, the Equality Office and the EaSE-2020 Fund); DAAD PPP Frankreich (Project-ID 55976814); the Hans-Böckler foundation; Ministère de l’Europe et des Affaires Etrangères (MEAE) and Ministère de l’Enseignement Supérieur, de la Recherche et de l’Innovation (MESRI; PHC PROCOPE 2017, Project no. 37733UM).

## Conflict of Interest

The authors declare that the research was conducted in the absence of any commercial or financial relationships that could be construed as a potential conflict of interest.

## Publisher’s Note

All claims expressed in this article are solely those of the authors and do not necessarily represent those of their affiliated organizations, or those of the publisher, the editors and the reviewers. Any product that may be evaluated in this article, or claim that may be made by its manufacturer, is not guaranteed or endorsed by the publisher.

## Abbreviations

3D LSM, Three-dimensional laser scanning microscopy; AHL, Acyl homoserine lactone; CV, Crystal violet; CD, β-cyclodextrin; CF, Cystic fibrosis; CF isolates, *Pseudomonas aeruginosa* clinical isolates from cystic fibrosis patients; CPM, Counts per minute; Ctr, Control; cGMP, Cyclic guanosine monophosphate; DMSO, Dimethyl sulfoxide; E_2,_ Estradiol; EPS, Extracellular polymeric substance; FE-SEM, Field emission scanning electron microscopy; h, hour; OMV, Outer membrane vesicles; QS, Quorum sensing; RT, Room temperature; R_t,_ Roughness height; s, seconds; Sq, Average roughness; WSE_2,_ Water soluble estradiol.
