# Supplementary material for: Effects of the Sex Steroid Hormone Estradiol on Biofilm Growth of Cystic Fibrosis Pseudomonas aeruginosa Isolates
Source: Front Cell Infect Microbiol. 2022 Jul 13;12:941014. doi: 10.3389/fcimb.2022.941014 (PMC9326073; doi:10.3389/fcimb.2022.941014)
Supplement: Supplementary file 1 [file DataSheet_1.pdf]

Supplementary Material

Supplementary Table 1 List of bacteria used in this study

Strains/Isolates		Source	Provider/place of isolation (Reference)
**Non-CF strains**			
1	PAO1	Skin wound	ATCC/ Rockville, in 1954 from a wound in Melbourne, Australia (Holloway, 1955)
2	ATCC19660 ™	Sepsis	ATCC/ Lima, Peru (Rosenthal, 1967)
**Clinical isolates from CF patients (respiratory infection)**			
1	PAH	CF (sputum)	CF-Reference center Nantes-Roscoff/ INSERM/CHRU Brest, FR (Berchel et al., 2011; Le Gall et al., 2013; Mottais et al., 2018)
2	C21-C2	CF (sputum)	CF-Reference center Nantes-Roscoff/ INSERM/CHRU Brest, FR
3	C4-C1	CF (sputum)	CF-Reference center Nantes-Roscoff/ INSERM/CHRU Brest, FR
4	CH2678	CF (sputum)	Charité Berlin [B]
5	M70565254	CF (pharyngeal)	University Hospital Münster [B]
6	MHH0985	CF (nd)	Hannover Medical School [B]
7	MHH15204	CF (nasopharyngeal)	Hannover Medical School [B]
8	MHH16563	CF (nd)	Hannover Medical School [B]
9	MHH17767	CF (tracheobronchial secretion)	Hannover Medical School [B]
10	MHH2419	CF (nd)	Hannover Medical School [B]

[B]: isolate collection kindly provided by Susanne Häußler, Helmholtz Centre for Infection Research, Braunschweig, Germany; further information about clinical CF isolates in Supplementary Table 2, Supplementary Figure 1-2 and these studies: (Hornischer et al., 2019; Khaledi et al., 2020; Thöming and Häussler, 2022); nd: not defined; ATCC: American Type Culture Collection, CHRU: University teaching hospital in Brest; INSERM: Institute national de la santé et de la recherche médicale.

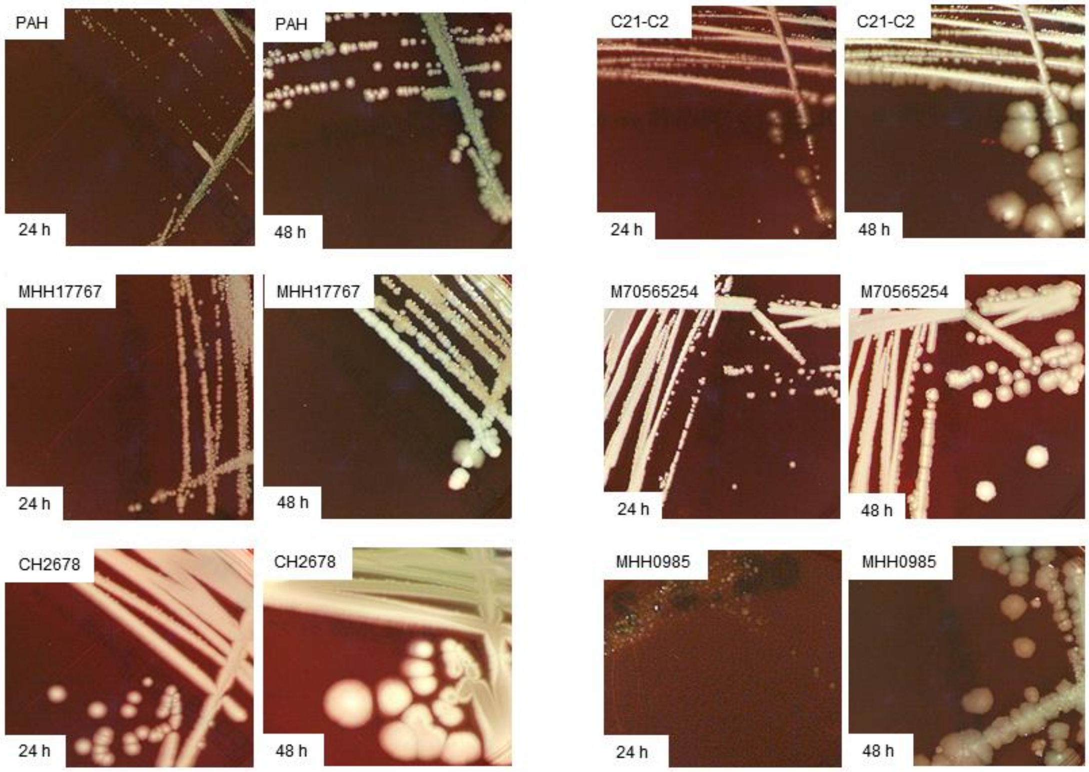

Supplementary Figure 1 Colony morphology of tested CF isolates and non-CF strains. Description see next page.

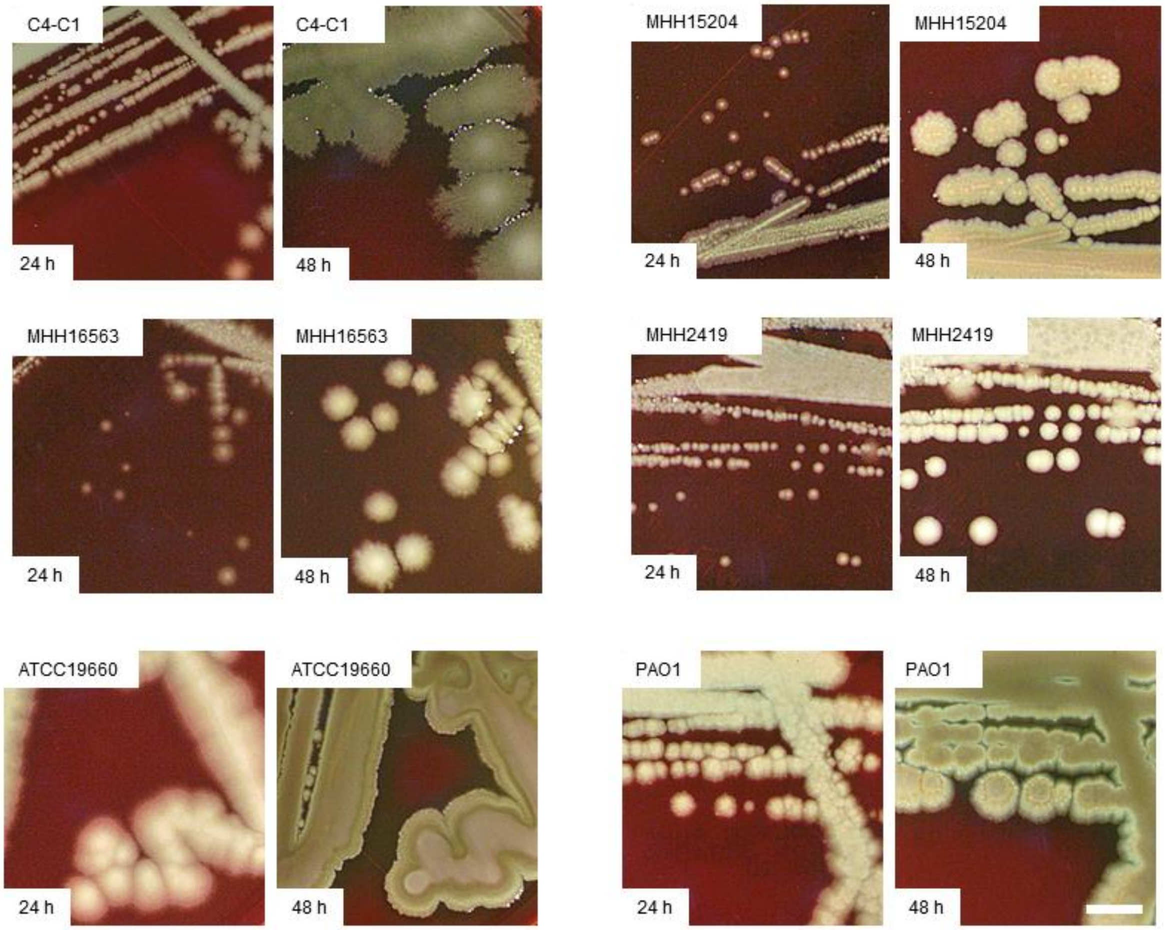

Supplementary Figure 2 Colony morphology of tested CF isolates and non-CF strains. *P. aeruginosa* non-CF strains and CF isolates were grown on blood agar plates for 24 h and 48 h at 37 °C after streaking them from glycerol stocks. Standardized documentation was done after an incubation time of 24 h and 48 h via a self-build black box with integrated USB camera. Isolates show marked differences in morphology in terms of size, form, surface appearance but also color. Scale bar: 12 mm.

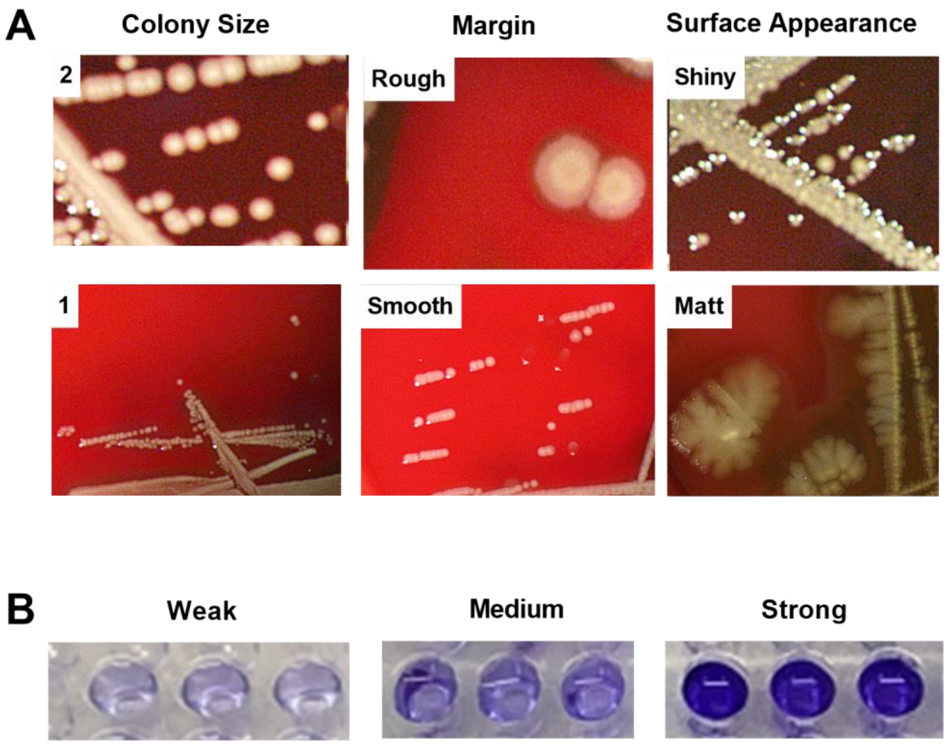

Supplementary Figure 3 Complementary illustration for categorization in Supplementary Table 2 of the morphological properties of CF isolate colonies grown on blood agar plates (**A)** and attached biofilms after crystal violet (CV) staining and dissolving in EtOH (**B**). A: Morphology of the isolates was determined based on three categories: colony size (small colonies = 1, big colonies = 2), colony margin (smooth/ sharp border, rough/ fringed border) and surface appearance (shiny and matt). B: Biofilms were grown under static conditions at 37 °C for 48 h in 96 U bottom PVC well plates. Attached biofilms were stained with CV and dissolved in 95% EtOH. The intensity of purple stain is proportional to the biofilm biomass, resulting in the following categories of biofilm formation from left to right; weak < medium < strong.

Supplementary Table 2 Additional information about morphotype and antibiotic resistance of CF isolates and lab strains after 24 h growth on blood agar plates (compare Supplementary Figure 1, 2)

CF isolate/non-CF strain	Colony Size (24 h)	Margin (24 h)	Biofilm formation	Surface appearance (24 h- 48 h)	TOB	CIP	MEM	CAZ	COL
**E2 responsive CF-isolates**									
PAH	1	smooth	weak	shiny	R	R	I	R	R
C21-C2	1	rough	weak	matt	R	R	R	R	S
MHH17767	1	smooth	medium	shiny	R	S	R	R	S
M70565254	1	smooth	medium/strong	matt/shiny	S	S	R	R	R
CH2678	2	smooth	strong	matt	S	R	S	S	R
MHH0985	1	rough	weak	shiny	I	I	R	R	S
**Non-responsive CF isolates**									
C4-C1	2	rough	strong	matt	S	S	S	S	S
MHH15204	2	rough	medium	matt	S	I	R	S	S
MHH16563	2	rough	medium/ strong	matt	S	S	I	I	S
MHH2419	2	smooth	strong	matt	S	S	S	S	S
**Non-CF strains**									
PAO1	2	rough	strong	matt	nd.				
ATCC19660	2	rough	strong	matt	nd.				

TOB; Tobramycin, CIP: Ciprofloxacin; MEM = Meropenem; CAZ = Ceftazidime; COL = Colistin; R (red): Resistant; I (yellow): Intermediate; S (green): Susceptible; nd: not defined; 1: category of small colonies; 2: category of bigger colonies. For categorization, see annotations in Supplementary Figure 2.

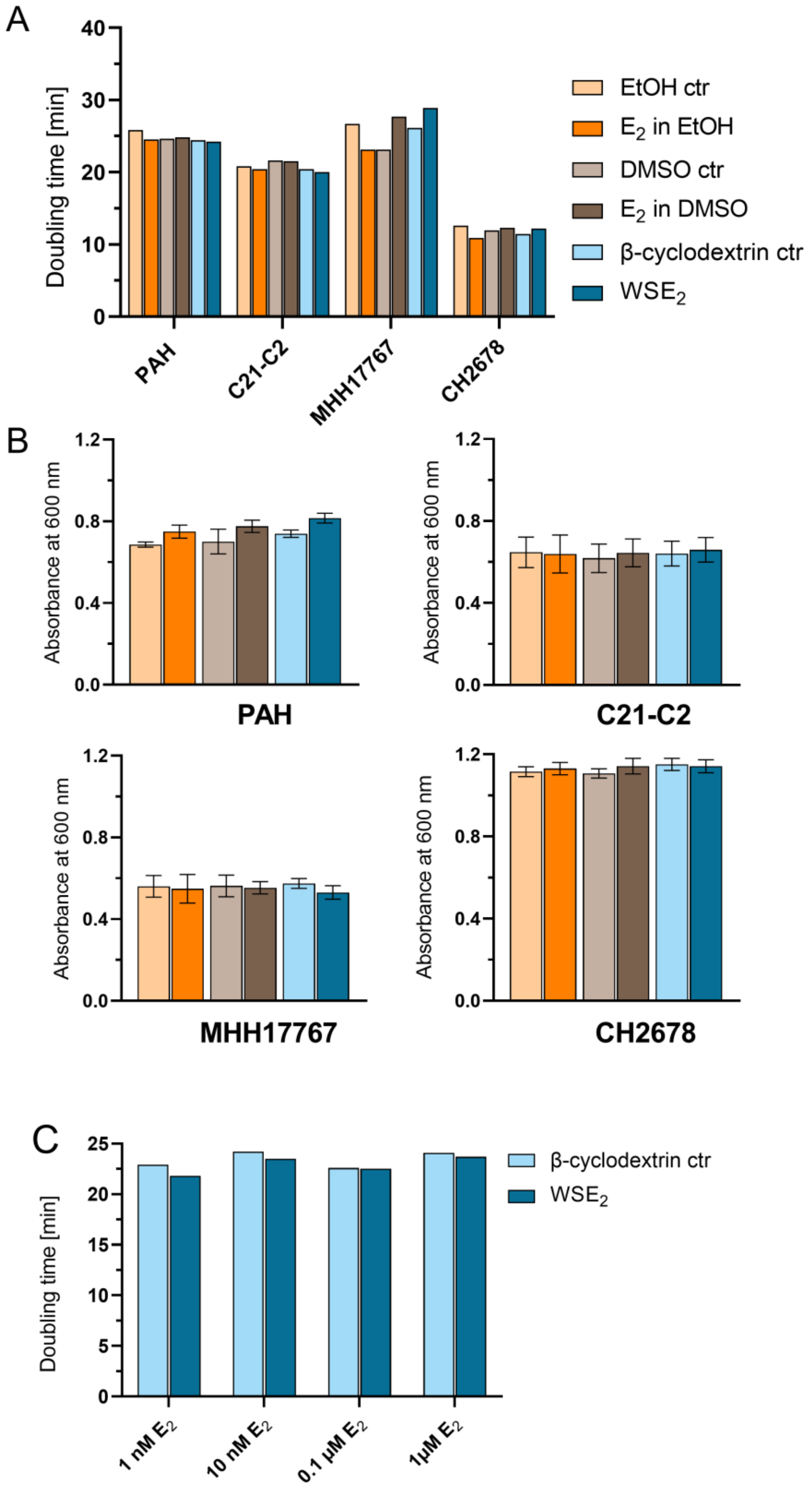

Supplementary Figure 4 E_2 does not have any essential effect on planktonic growth of E_2 responsive CF isolates. CF isolates PAH, C21-C2, MHH17767 and CH2678 were grown in 96-well transparent flat bottom plate with lid (Sarstedt AG & Co. KG, Nürnbrecht, Germany) for 24 h and

37°C measuring in parallel absorbance at OD600 with a kinetic interval of 10 min in a multiplate reader. CF isolates were treated with E_2 and corresponding solvent control conditions. Measurements were performed in triplicates (3 wells). Planktonic growth was characterized via calculation of the doubling times based on the exponential phase of the recorded growth curve (**A**) and total absorbance (OD_{600}) at the endpoint of the 24 h growth (**B**). Error bars: Standard deviation (n = 3) **C:** Doubling time of PAH in dependance of WSE_2 concentrations in the culture ranging from 1 nM to 1 µM. As solvent adjuvants control the corresponding β-cyclodextrin concentrations ranging from 5 nM to 50 µM was applied.

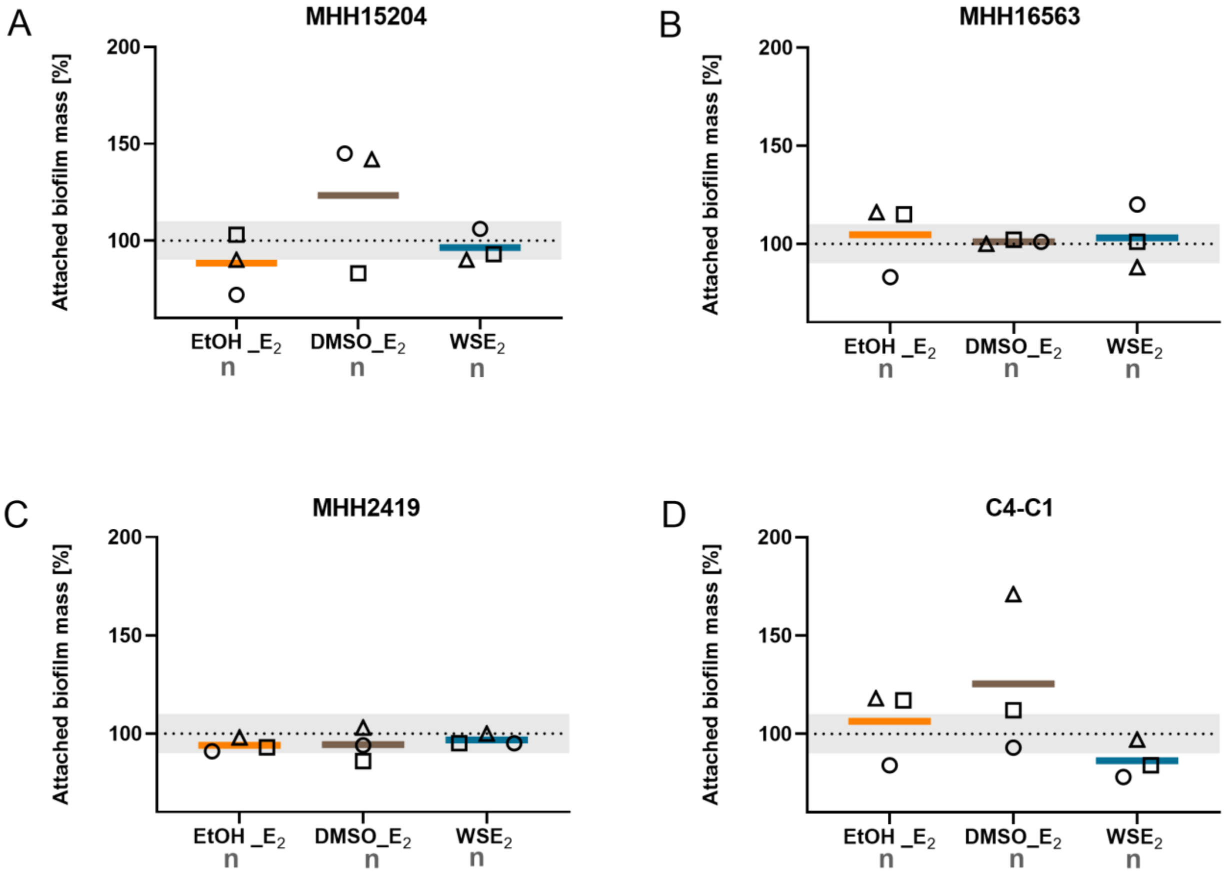

Supplementary Figure 5 Non-E_2 responsive CF isolates regarding attached biofilm mass. *P. aeruginosa* isolates MHH15204 (**A**), MHH16563 (**B**), MHH2419 (**C**) and C4-C1 (**D**) were treated with 10 nM E_2 in EtOH (orange), E_2 in DMSO (brown), WSE_2 (blue), or corresponding solvent controls (0.0001 % v/v EtOH, 0.00001 % v/v DMSO or 50 nM β-cyclodextrin in H_2O) for 48 h. Attached biofilm mass was quantified via CV assay. Solvent controls were set to 100 % (see dotted line). Colored horizontal lines indicate mean values of 3 independent experiments (○ = experiment 1, □ = experiment 2, △= experiment 3) with each single experiment based on at least 6 technical replicates. E_2 responsiveness was defined as more than 10% change (indicated by grey zones between 90% and 110%). n: no effect (see materials/methods for detailed description).

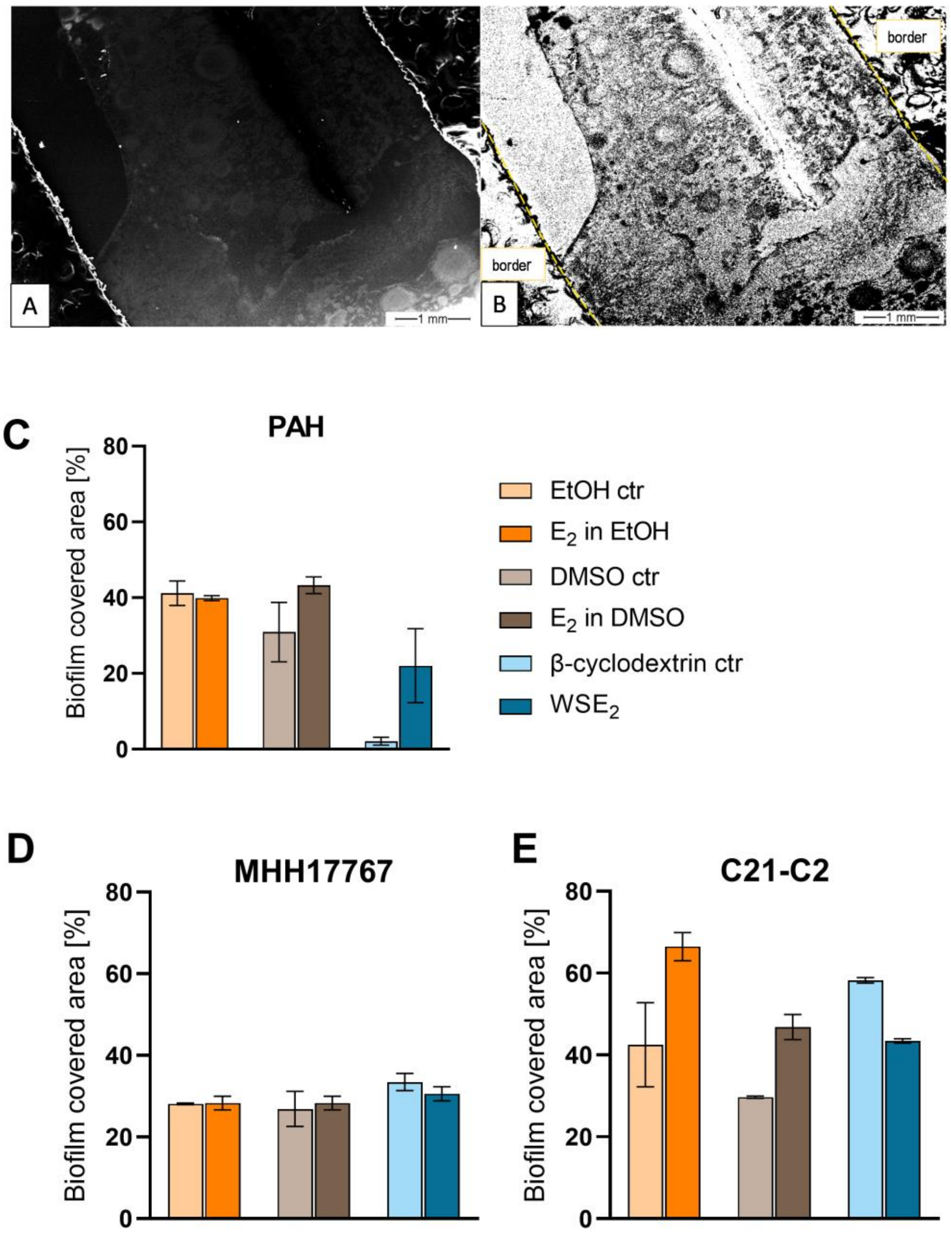

Supplementary Figure 6 Quantification of biofilm covered area based on SEM images of CF isolates treated with E_2. CF isolates PAH (**C**), MHH17767 (**D**) and C21-C2 (**E**) were treated with 10 nM E_2/ WSE_2, or corresponding solvent controls. The samples were analyzed via SEM (CamScan 24 SEM, West Orange, USA) with an applied voltage of 25 kV at a 19-fold magnification to provide an overview of the sample. Working distance was between 26 mm and 33 mm and had to be adjusted due to the slight curvature of the samples. For the quantification of the area covered with biofilm SEM pictures (**A**) were analyzed via ImageJ. The obvious borders of the PVC substrate (yellow lines) were excluded from the analysis. The scale was set to 171 pixels/mm and a background correction was performed by the software (substract background, rolling ball radius set to 50.0 pixels). Brightness and contrast were adjusted to enhance visibility of the biofilm and the image type was set to 8-bit. The threshold was manually adjusted until the whole biofilm was marked in black

(**B**). This area was quantified via the function 'analyze particles': size (mm^2) = 0.10 – infinity, Circularity: 0.00 – 1.00. **C-E** show the mean coverage area [%] of 2 selected SEM areas and error bar indicates upper and lower value. Compare also other analyses of the same samples in Figures 4-7 and Supplementary Figures 7, 8 and 9.

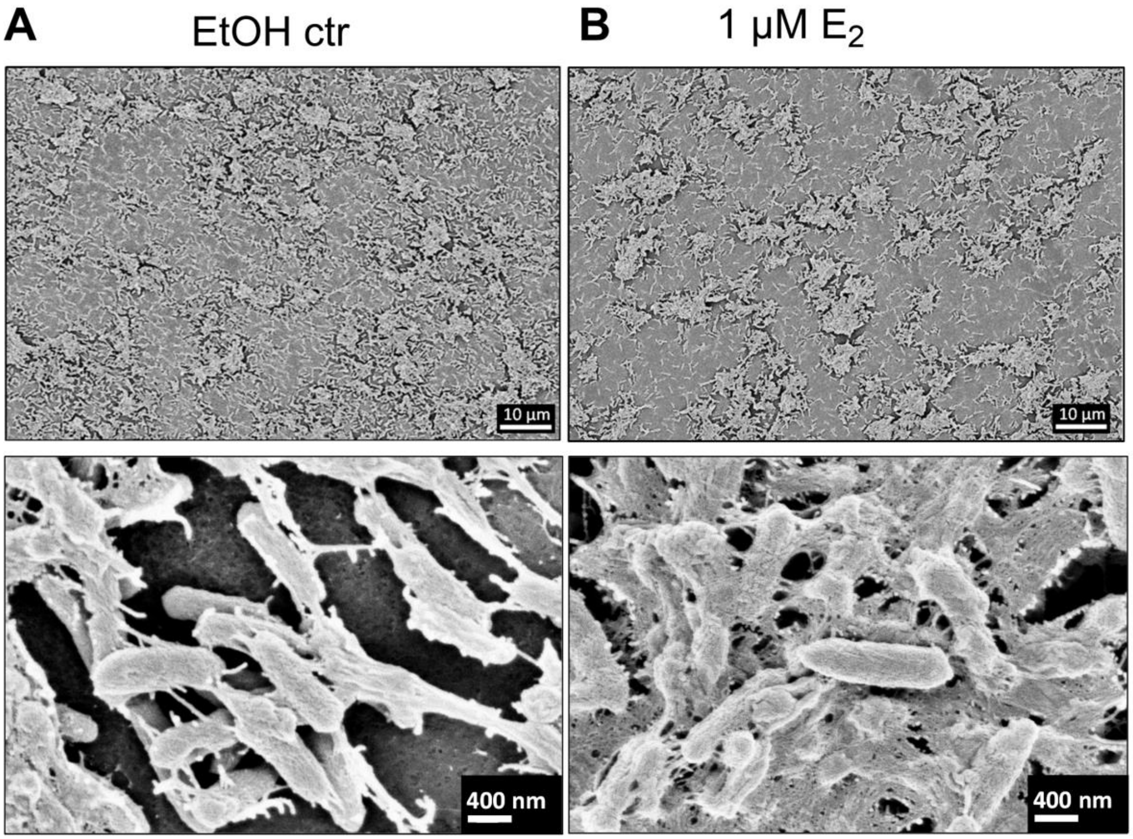

Supplementary Figure 7 FE-SEM images of PAH biofilms treated with E_2 show more clustering of bacteria towards multilayered microcolonies compared to control. PAH was grown for 48 h on polyethylene terephthalate transwell inserts (1.1 cm^2, Pore size: Ø 0.4 µm, Sarstedt, Nürnbrecht, Germany) in the presence of corresponding EtOH solvent control (**A**) and 10^{-6} M E_2 in EtOH (**B**). Upper images show overview of the biofilm structure indicating distribution of microcolonies and bacteria in between, lower pictures show higher magnifications visualizing single bacteria embedded in a complex network of extracellular polymeric substance (EPS). Scale bar: 10 µm (upper images), 400 nm (lower images).

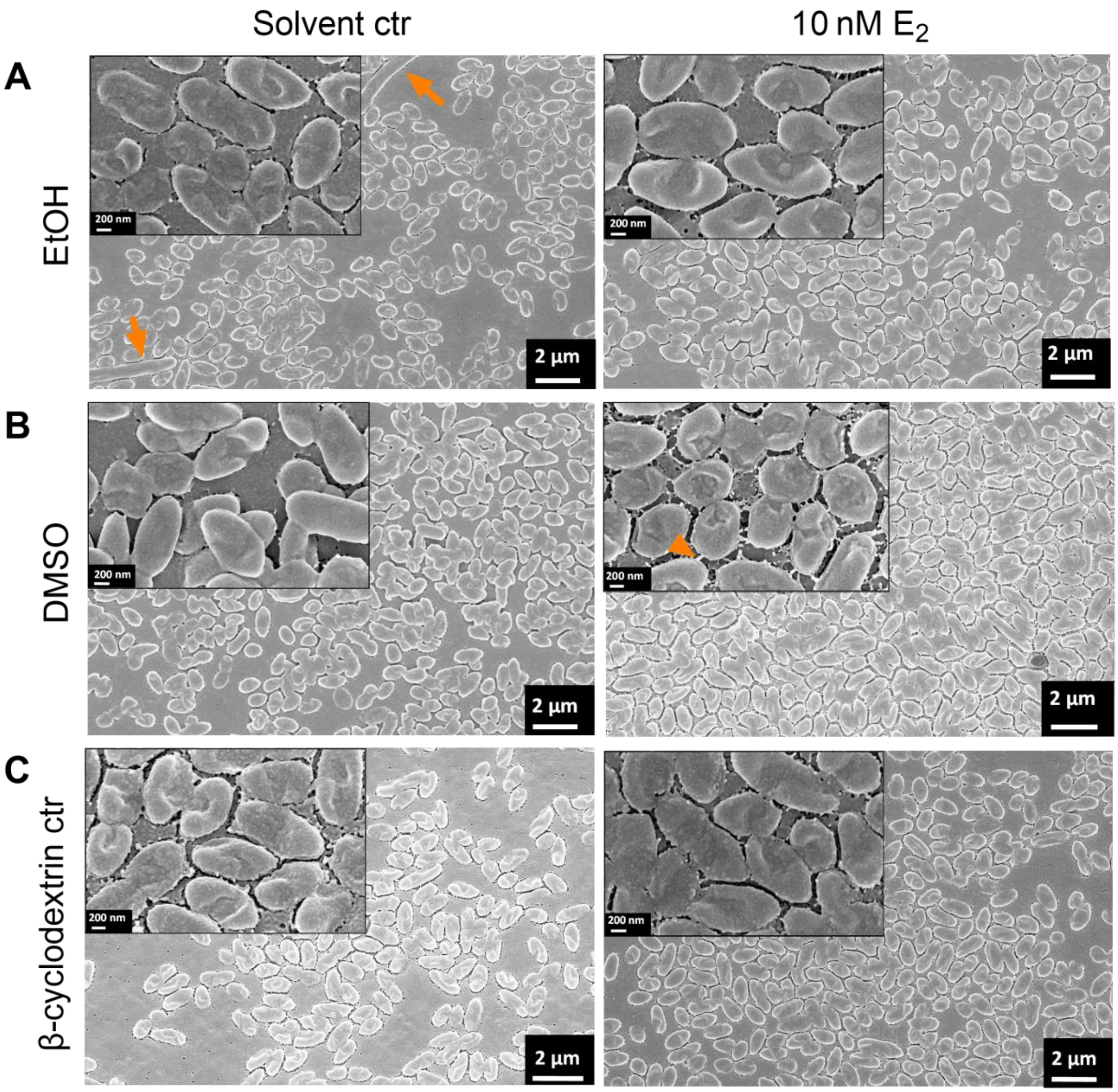

Supplementary Figure 8 Ultrastructural changes of C21-C2 biofilms after treatment with E_2. C21-C2 biofilms were grown with 10 nM E_2 in EtOH (**A**, right), E_2 in DMSO (**B**, right), WSE_2 (**C**, right) or the corresponding solvent control (0.0001% v/v EtOH, 0.00001% v/v DMSO or 50 nM β-cyclodextrin in H_2O) (**A**-**C** left). Exemplary FE-SEM pictures show biofilm areas close to air-liquid interface (max. 500 µm below). Image sections show higher magnification to illustrate the ultrastructure of the extracellular gaps between bacteria. Arrows in **A** (right) indicate elongated cells (Yoon et al., 2011). Arrowhead in **B** (section on the right) indicate extracellular bacterial appendages like amyloid fibrils, cup fimbriae or pili as components of the EPS (Wei and Ma, 2013; Rouse et al., 2018), scale bar: 2 µm, 200 nm (magnified sections).

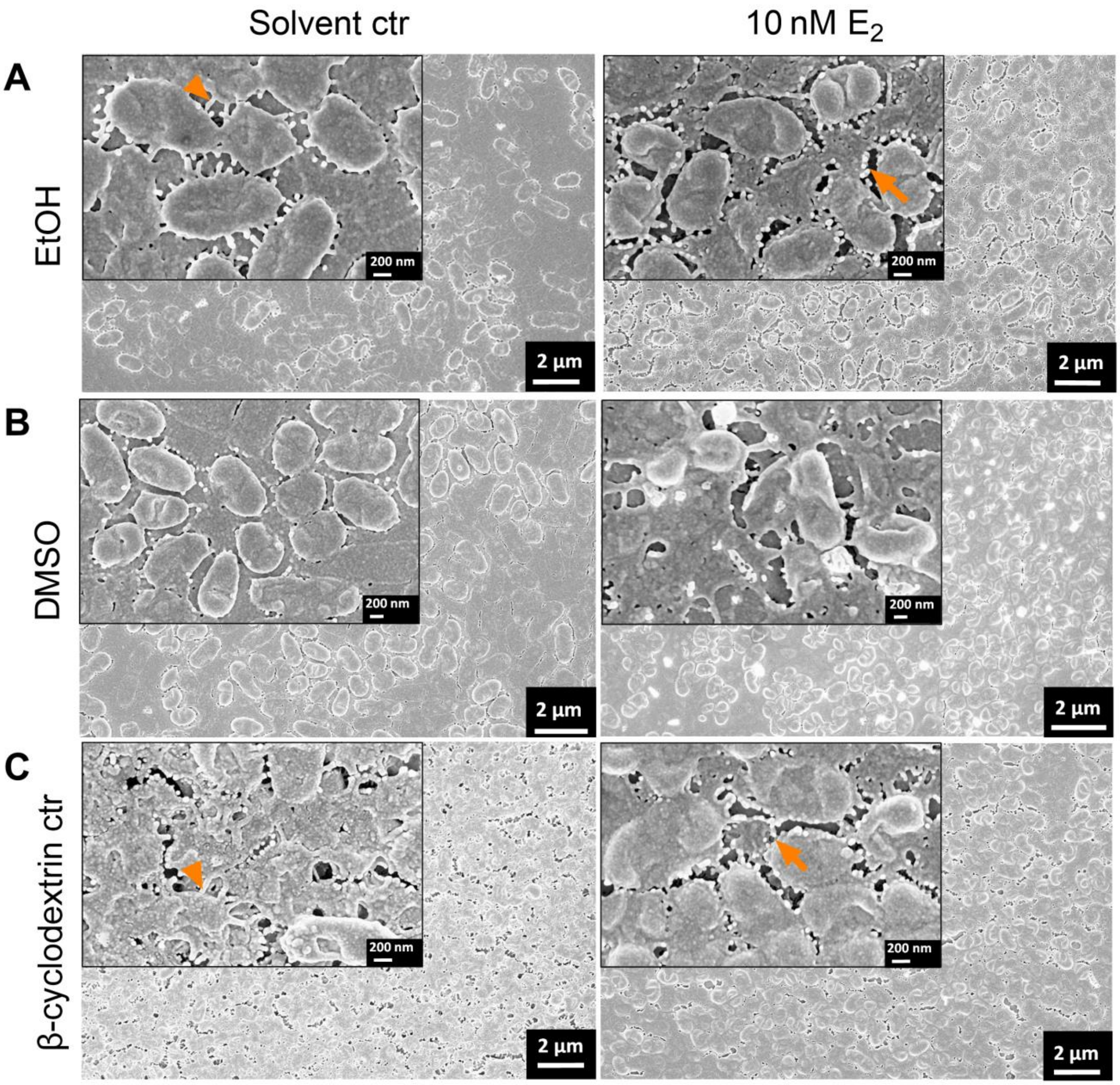

Supplementary Figure 9 Ultrastructural changes of non-CF strain PAO1 biofilms treated with E_2 (compare to experiments shown in Figure 4B ☐**).** PAO1 biofilms have been grown with 10 nM E_2 in EtOH (**A**, right), E_2 in DMSO (**B**, right), WSE_2 (**C**, right) or the corresponding solvent control conditions (**A-C**, left) (0.0001% v/v EtOH, 0.00001% v/v DMSO or 50 nM β-cyclodextrin). Exemplary FE-SEM pictures show biofilm areas close to the air-liquid interface (max. 500 µm below). Image sections show highest magnification to illustrate the ultrastructure of the extracellular matrix composition. Orange Arrowheads in **A** and **C** (left side section) indicate cell-cell connection that seem to be degenerated upon E_2 treatment (**A** and **C**, right section) ending up in prominent intercellular gaps between bacteria containing more outer membrane vesicles (Jan, 2017) (orange arrow in **A**, section on the right). Scale bar: 2 µm, 200 nm (magnified sections).

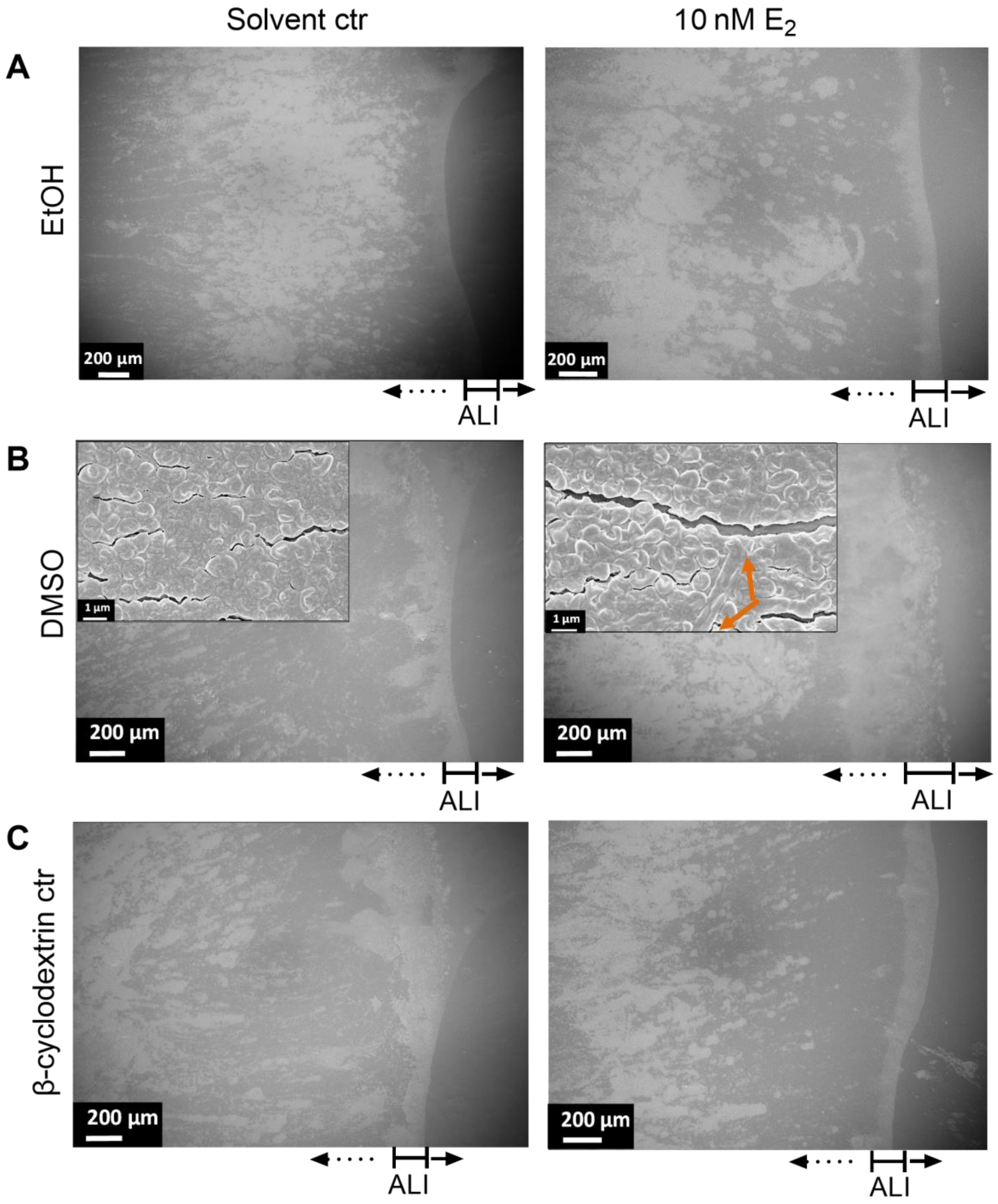

Supplementary Figure 10 Ultrastructural changes of CF isolate MHH17767 biofilms treated with E_2 (compare to experiments shown in Figure 2 DO). MHH17767 biofilms have been grown with E_2 in EtOH (**A**, right), E_2 in DMSO (**B**, right), WSE_2 (**C**, right) or the corresponding solvent control conditions (0.0001% v/v EtOH, 0.00001% v/v DMSO or 50 nM β-cyclodextrin). FE-SEM pictures show biofilm areas close to the air-liquid interface (ALI) (max. 500 µm below). The ALI is indicated by black arrows pointing to air (upper part of the well plate), dotted arrow is pointing to the submerged part of the biofilm (bottom of the well plate). In B inserts show magnifications of the biofilm at the ALI area. Orange arrows: elongated cell. Scale bar: 200 µm.

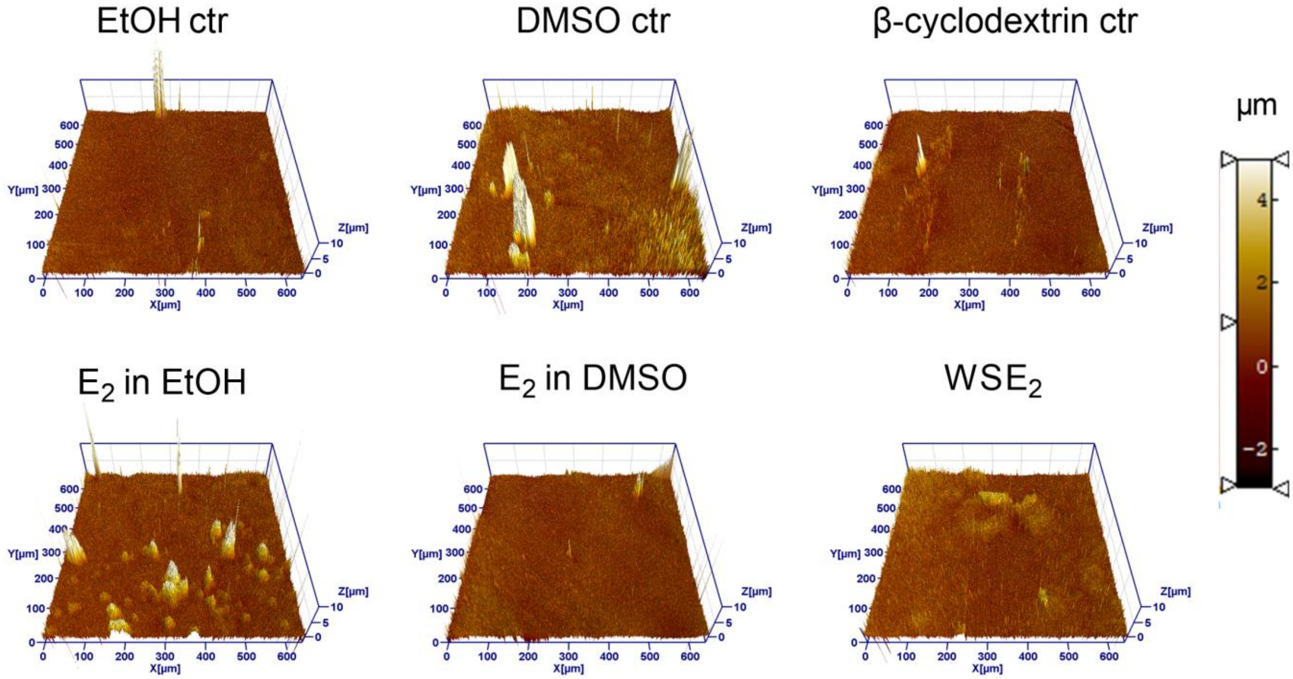

Supplementary Figure 11 3D LSM images of PAH biofilms upon E_2 treatment (compare zoom in 3D LSM pictures in Figure 7 and FE-SEM analysis in Figure 5 and 6 of the same sample).

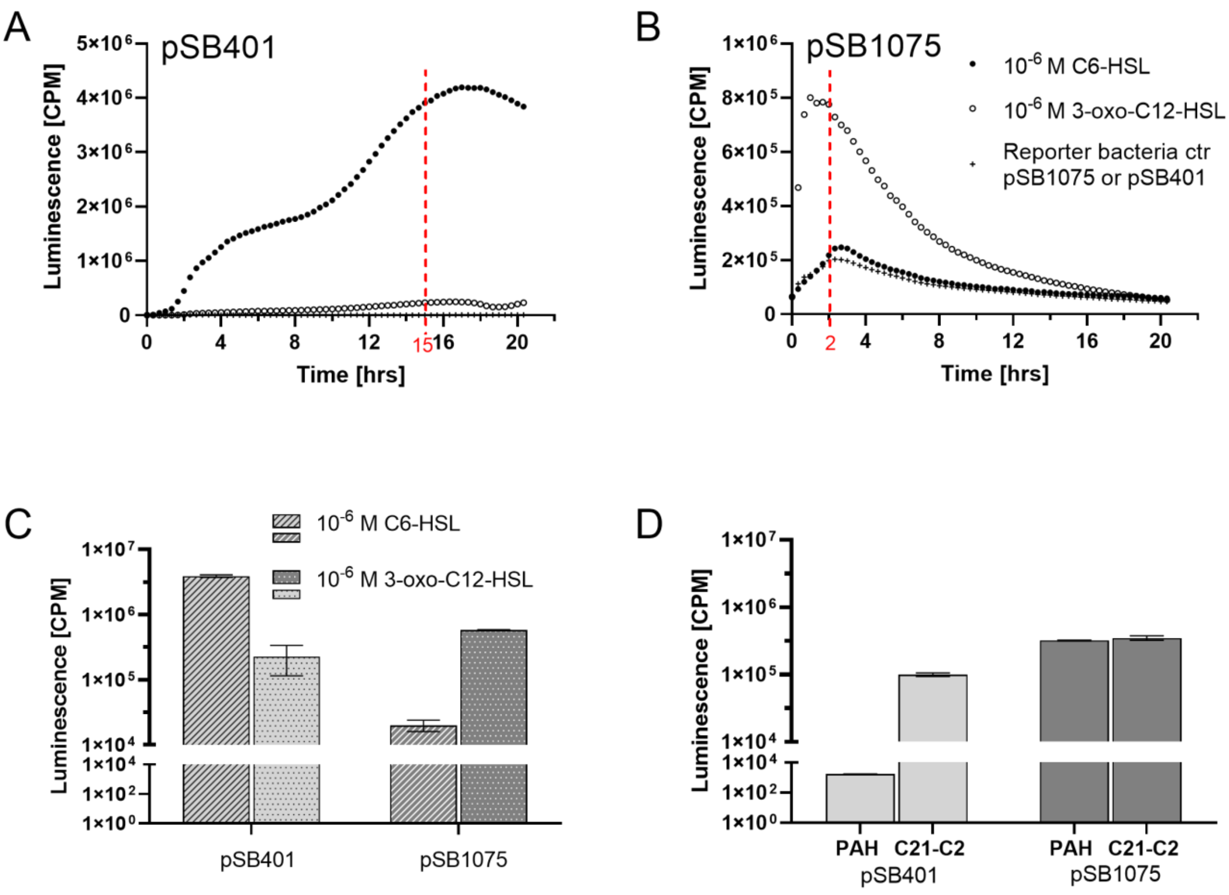

Supplementary Figure 12 AHL reporter assay performance and QS activity of PAH and C21-C2. The synthetic autoinducers C_6-HSL (N-Hexanoyl-DL-homoserine lactone) and 3-oxo-C_{12}-HSL (N-(3-Oxododecanoyl)-L-homoserine lactone) at a concentration of 10^{-6} M were measured by the luminescent reporter bacteria *E. coli* pSB401 (**A**) and pSB1075 (**B**). The reporter bacteria ctr reflects the background signal (noise without addition of AHLs or bacterial supernatants). The readout timepoints 15 h (for pSB401) and 2 h (for pSB1075) were chosen for further data analysis (red dotted line), which are plotted accordingly in a bar graph (**C**) to illustrate that both QS reporter bacteria result in luminescence increase after AHL administration when subtracting background signal (reporter bacteria ctr without AHLs). *E. coli* pSB401 seems to be more responsive to short chain AHLs like C_6-AHL and pSB1075 more responsive to long chain AHLs like 3-oxo-C_{12}-AHL. **D**: Illustration of QS activity in supernatants of untreated PAH and C21-C2 biofilms to illustrate different QS activity in the investigated CF isolate that hint to a diverse composition of AHLs in the supernatants of CF isolates. Error bars: standard deviation of 3 technical replicates, logarithmic y-axes in C and D.

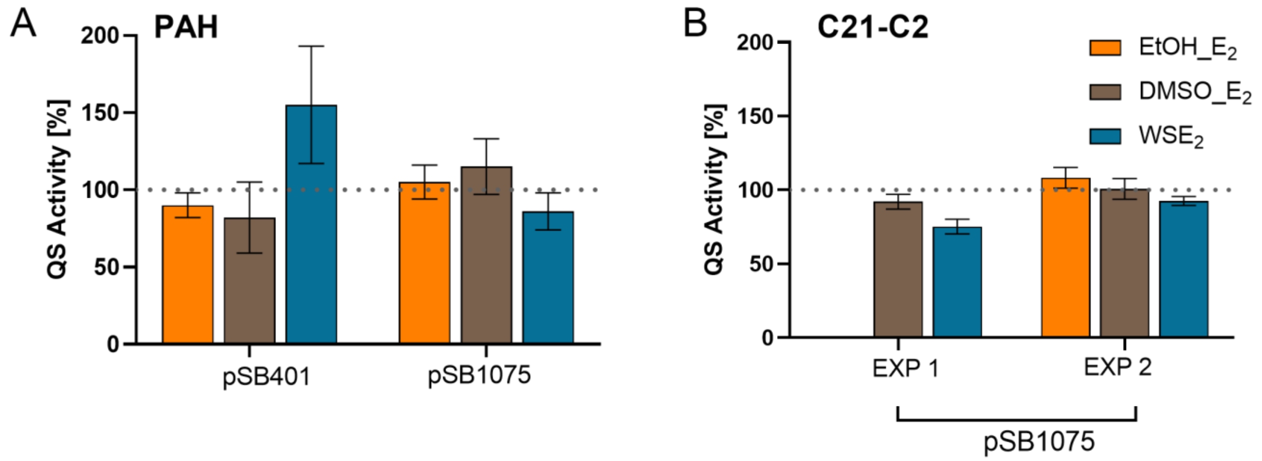

Supplementary Figure 13 QS activity in sterile supernatants of PAH and C21-C2 biofilms by E_2 (compare to experiment shown in Figure 2 B▽). Increase of QS activity [%] was calculated by dividing the luminescence increase (compare Supplementary Figure 12C and D) of sterile supernatants of biofilms treated with E_2 in EtOH, E_2 in DMSO or WSE_2 by the corresponding solvent control and multiplied this ratio with 100. QS activity under solvent control conditions was set to 100% (dotted line) **A**: QS activity of PAH biofilm supernatants were measured with both reporter bacteria pSB401 and pSB1075, reflecting a clear upregulation of pSB401 measured QS activity after WSE_2 treatment. **B**: QS activity of C21-C2 biofilm supernatant collected from 2 independent E_2 biofilm treatments experiments (EXP1/EXP2) was measured by pSB1075. Here, C21-C2 supernatants showed only a higher QS activity when treated with E_2 in EtOH compared to the control. Error bars: Standard deviation of 3 technical replicates.

References

Berchel, M., Gall, T. Le, Denis, C., Hir, S. Le, Quentel, F., Elléouet, C., et al. (2011). A silver-based metal-organic framework material as a “reservoir” of bactericidal metal ions. *New J. Chem.* 35, 1000–1003. doi:10.1039/c1nj20202b.

Holloway, B. W. (1955). Genetic recombination in *Pseudomonas aeruginosa*. *Microbiology* 13, 572–581. doi:10.1099/00221287-13-3-572.

Hornischer, K., Khaledi, A., Pohl, S., Schniederjans, M., Pezoldt, L., Casilag, F., et al. (2019). BACTOME - a reference database to explore the sequence- and gene expression-variation landscape of *Pseudomonas aeruginosa* clinical isolates. *Nucleic Acids Res.* 47, D716–D720. doi:10.1093/nar/gky895.

Jan, A. T. (2017). Outer Membrane Vesicles (OMVs) of gram-negative bacteria: A perspective update. *Front. Microbiol.* 8, 1–11. doi:10.3389/fmicb.2017.01053.

Khaledi, A., Weimann, A., Schniederjans, M., Asgari, E., Kuo, T., Oliver, A., et al. (2020). Predicting antimicrobial resistance in *Pseudomonas aeruginosa* with machine learning-enabled molecular diagnostics. *EMBO Mol. Med.* 12: e10264. doi:10.15252/emmm.201910264.

Le Gall, T., Berchel, M., Le Hir, S., Fraix, A., Salaün, J. Y., Férec, C., et al. (2013). Arsonium-

containing lipophosphoramides, poly-functional nano-carriers for simultaneous antibacterial action and eukaryotic cell transfection. *Adv. Healthc. Mater.* 2, 1513–1524. doi:10.1002/adhm.201200478.

Mottais, A., Berchel, M., Sibiril, Y., Laurent, V., Gill, D., Hyde, S., et al. (2018). Antibacterial effect and DNA delivery using a combination of an arsonium-containing lipophosphoramide with an N-heterocyclic carbene-silver complex – Potential benefits for cystic fibrosis lung gene therapy. *Int. J. Pharm.* 536, 29–41. doi:10.1016/j.ijpharm.2017.11.022.

Rosenthal, S. M. (1967). Local and systemic therapy of *pseudomonas* septicemia in burned mice. *Ann. Surg.* 165, 97–103. doi:10.1097/00000658-196701000-00013.

Rouse, S. L., Matthews, S. J., and Dueholm, M. S. (2018). Ecology and Biogenesis of Functional Amyloids in *Pseudomonas*. *J Mol Biol*. 12;430(20):3685–3695. doi: 10.1016/j.jmb.2018.05.004.

Thöming, J. G., and Häussler, S. (2022). *Pseudomonas aeruginosa* is more tolerant under biofilm than under planktonic growth conditions: A Multi-Isolate Survey. *Front. Cell. Infect. Microbiol.* 12, 1–10. doi:10.3389/fcimb.2022.851784.

Wei, Q., and Ma, L. Z. (2013). Biofilm matrix and its regulation in *Pseudomonas aeruginosa*. *Int. J. Mol. Sci.* 4(10), 20983–21005. doi:10.3390/ijms141020983.

Yoon, M. Y., Lee, K. M., Park, Y., and Yoon, S. S. (2011). Contribution of cell elongation to the biofilm formation of *Pseudomonas aeruginosa* during anaerobic respiration. *PLoS One* 6, 1–11. doi:10.1371/journal.pone.0016105.
